# Standard deviation of pulse pressure measured using wearable devices improves the estimation of acute psychological stress

**DOI:** 10.1038/s41598-025-24704-2

**Published:** 2025-11-20

**Authors:** Tomohisa Shiotani, Masayuki Minakata, Eiji Toyoda, Chiharu Odane, Uday Mitsuyasu, Takashi Nakao

**Affiliations:** 1 Marketing Division, Digital Health Promotion Department, Nitto Denko Corporation, 1-1-2 Shimohozumi, Ibaraki-shi, Osaka, Japan; 2 Social Care Division, Digital Health Promotion Department, Nitto Denko Corporation, 1-1-2 Shimohozumi, Ibaraki-shi, Osaka, Japan; 3 Graduate School of Human and Social Sciences, Hiroshima University, 1-1-1 Kagamiyama, Higashihiroshima-shi, Hiroshima, Japan

**Keywords:** Pulse pressure, Standard deviation of pulse pressure, Stress monitoring, Wearable device, Trier social stress test, Heart rate, Heart rate variability, Salivary cortisol, Psychology, Predictive markers, Psychophysics

## Abstract

Recent technical innovations have increased the use of physiological information as objective indices for stress measurement. Early detection of acute stress can minimize long-term stress burdens by aiding in the prevention and treatment of stress-related physical and mental health problems. The standard deviation of pulse pressure (SDPP) has recently emerged as an objective index for measuring stress. However, the causal relationship between acute stress and SDPP has not been verified, and it is unclear whether SDPP can detect stress responses that conventional indices cannot. This study investigated whether SDPP, measured using a wearable device, can be used as a stress assessment index to detect acute psychological stress and improve the accuracy of stress estimation in healthy participants who were subjected to the trier social stress test (TSST). A total of 114 healthy volunteers were randomly divided into the stress- induced (Stressed) and non-stress (Control) groups. Heart rate (HR) and heart rate variability (HRV), which are indices of the autonomic nervous system, and salivary cortisol, an endocrine marker, were used as objective stress indices. Psychological evaluations (POMS2, STAI) were employed as subjective assessments. The relationships between these measurements and SDPP were evaluated. The results indicated that SDPP was significantly higher in the Stressed group, confirming that SDPP reflects induced stress. Furthermore, logistic regression analysis showed that adding SDPP to HR, HRV, and cortisol can improve the accuracy of estimating stress presence. Moreover, a multilevel analysis revealed that SDPP can enhance the estimation of psychological evaluation (POMS [total mood disturbance]) scores. The results suggest that SDPP can estimate the degree of stress experienced by an individual and monitor stress responses undetectable by conventional indices.

**Supplementary Information:**

The online version contains supplementary material available at 10.1038/s41598-025-24704-2.

## Introduction

In modern society, individuals are exposed to various unavoidable stressors due to mental and physical demands. Chronic stress is considered an indirect contributor of lifestyle-related diseases, such as obesity, primarily through its disruption of lifestyle habits. Given its impact on health, there is an increasing need for methods that can monitor acute stress, which can become the precursor to chronic stress. Because stress is known to cause changes in the autonomic nervous system and the endocrine system^1^, numerous studies have explored their relationships with acute stress^2–5^.

The analysis of the autonomic nervous system typically involves data obtained from ECG, or pulse wave signals derived from photoplethysmogram (PPG)^6–8^. Autonomic nervous system indicators each have their own strengths and weaknesses, so it is recommended to use multiple indicators to assess stress^9^. Among these, the standard deviation of pulse pressure (SDPP) is an indicator that has recently begun to attract attention and shows potential for capturing autonomic nervous system responses associated with stress^10^. However, research on stress assessment using SDPP is currently extremely limited. It is unknown whether SDPP changes due to acute stress and whether it can capture responses that are difficult to detect using conventional autonomic nervous system indicators. Therefore, this study aims to clarify changes in SDPP induced by acute stress and to evaluate its utility by comparing it with conventional autonomic nervous system indicators.

First, we will clarify the conventional indicators used for comparison in this study: heart rate (HR) and heart rate variability (HRV). Analyses of data obtained from electrocardiograms (ECGs) and pulse wave information obtained from photoplethysmography (PPG) devices traditionally include HR and HRV13. HR is a fundamental indicator for assessing the state of the heart and the entire body. When stress is applied, the sympathetic nervous system (SNS) becomes activated, causing HR to increase14. On the other hand, HRV reflects the variability of the heartbeat cycle, and analysis methods such as time-domain analysis and frequency-domain analysis exist ^11,12^.Numerous studies have been conducted on methods for analyzing mental stress using HRV^11–17^, and several real-time analysis applications have been proposed. However, HRV is highly sensitive to data quality and is heavily dependent on data filtering for the removal of erroneously detected peaks, as these errors can significantly distort the results. Furthermore, wearable devices are heavily prone to noise artifacts, and HRV based on these signals are often found to be unreliable. Because of these strengths and limitations of autonomic nervous system indices^18–20^, stress is best assessed using multiple indices^18^.

In light of these limitations, recent studies have explored stress estimation using blood pressure^21,22^. It has been reported that acute stress increases pulse pressure (PP), defined as the difference between systolic blood pressure (SBP) and diastolic blood pressure (DBP)^9,23,24^. Additionally, participants who underwent stress management training exhibited reduced SBP variability compared to those without training^25^, suggesting a close relationship between stress and blood pressure variability. Studies on PP variability have reported that exercise-induced SNS activation is reflected more strongly in the low-frequency power of SBP variability than in DBP variability. This is supported by multiple reports indicating that the low-frequency power of SBP variability may be influenced by the autonomic nervous system^26,27^. Furthermore, a strong correlation between the low-frequency power of SBP and PP variability suggests that PP variability may also reflect SNS activity^10^.

The relationship between the standard deviation of PP (SDPP) and stress was reported by Burnett-Zeigler et al.^28^. Burnett-Zeigler et al. studied adult women with mild to severe depression, measuring SDPP over a one-week period following a 30-day subjective assessment, using an experimental watch-type wearable device that non-invasively captured PPG signals to derive pulse wave information. The study reported a positive correlation between subjective chronic stress scores and SDPP. However, Burnett-Zeigler et al. did not manipulate stress to observe changes in SDPP, so it remains unclear whether SDPP responds to acute stress. Additionally, it is unclear whether SDPP can capture stress responses that cannot be detected by other objective stress indicators such as HR or HRV. If these relationships are established, SDPP, which can be measured using wearable devices, has the potential to improve the accuracy of acute psychological stress estimation in daily life.

Therefore, in this study, we examined whether SDPP can enhance the accuracy of acute stress estimation. We used the Trier Social Stress Test (TSST) to induce acute stress and measured SDPP using a device (Counseling System by Nitto Denko Corporation) similar to the one used by Burnett-Zeigler et al., which derives SDPP from non-invasive PPG measurements. This approach allowed us to confirm whether SDPP reflects stress. In addition, we determined whether SDPP reflects stress responses not detected by conventional stress indices. In other words, we assessed whether incorporating SDPP can improve the estimation of stress condition. As conventional acute stress indices, HR and HRV, which are indices of the sympathetic–adrenal medullary (SAM) system response, were measured using wearable devices (myBeat Heart Rate Sensor WHS-3; Union Tool Co., Tokyo, Japan). Because activity in the endocrine system is primarily analyzed by observing hormones and other markers in blood, saliva, and urine, cortisol, a representative marker of a different pathway from the SAM, namely the hypothalamic-pituitary-adrenocortical (HPA) system response, was also measured. Cortisol is a steroid hormone released by the adrenal cortex, and is the most frequently studied substance in relation to acute stress. Cortisol has various physiological effects on the immune, vascular, and central nervous systems and is an important hormone when considering psychological and physical health conditions^29^. Salivary amylase secretion is also commonly used in stress response research, but is associated with the SAM system, and exhibits psychological responses distinct from those of cortisol. In this study, cortisol was selected to examine whether the inclusion of HPA axis could improve the accuracy of estimating acute psychological stress using SDPP.

Three hypotheses were proposed in this research: (1) the SDPP value of the Stressed group will be greater than that of the Control group during the stress-inducing periods; (2) the addition of SDPP to HR, HRV, and cortisol will improve the differentiation between the Stressed and Control groups; and (3) the incorporation of SDPP will enhance the estimation of subjective mood changes associated with stress loading in the Stressed group.

## Methods

### Participants

A total of 114 (58 women and 56 men) healthy, Japanese-speaking participants from Tokyo, Japan, aged 21–58 years were recruited for the experiment. Anticipating the Mann–Whitney U test (*α* = 0.05, power = 0.9, effect size = 0.8) for the comparison of the mean SDPP between the Stressed group and the Control groups, the required sample size was estimated to be 40 participants per group through an a priori power analysis. The participants (*n* = 114) were randomly assigned to the Control group (*n* = 57) and the Stressed group (*n* = 57). The mean ages of the Control group and the Stressed group were 44.5 years (*SD* = 9.6) and 44.9 years (*SD* = 11.0), respectively. The gender distribution was the same between the groups, with 28 men (49%) and 29 women (51%) in each group. Two participants (4%) in the Control group and 5 (9%) in the Stressed group were smokers (Supplementary Information Table S1). All the participants were healthy enough to successfully participate in the experiment, and had no prior history of psychiatric issues.

TSST^30–32^ was used to induce stress on the participants. All experiments were conducted in accordance with the Declaration of Helsinki. The participants provided informed consent prior to participation, and all procedures were approved by the Shiba Palace Clinic Ethics Review Committee (Protocol No.: 152327–35175). Furthermore, the participants were given an explanation regarding the purpose and procedures of the study, particularly the psychosocial stress test, as well as the schedule of the experiments. However, they were not informed of the specific details of the TSST to prevent a decrease in stress response due to prior mental adaptation to the anticipated tasks. The collected data were anonymized and securely stored to prevent information leakage. To minimize the effect of diurnal variation in cortisol level, all tests were conducted between 1 and 8 pm.

During measurement and analysis, some missing data were observed for cortisol, HR, and HRV. For the analysis of cortisol, two participants (2%) for whom saliva measurements could not be performed were excluded as outliers. For HR, two participants (2%) with poor measurements were excluded. For HRV (low-frequency (LF)/high-frequency (HF), HF), an additional 10 participants (9%) were excluded using the preprocessing procedure described below. As some analyses did not include all sample data, the sample size for each analysis is reported in the table or table notes in the Results section and in the Supplemental Information.

To investigate the influence of participants (*n* = 16) who smoke, take medication, have poor health status, or have preexisting illness, we excluded them and developed a logistic regression model using the difference between the groups as the response variable. Then, we compared the data before exclusion with the data obtained immediately after stress manipulation and confirmed that there were no influences from these factors (Supplemental Information Table S9 and Table S10).

### Measurements

#### SDPP

For the SDPP measurement, a wristwatch-type wearable device from the Counseling System (Nitto Denko Corporation) was used for the collection and real-time visualization of SDPP. The participants wore the device on their nondominant wrist and sat in a chair, with their device-wearing arm secured to the armrest with a strap. The original TSST protocol requires standing, but since standing or changes in posture can cause fluctuations in blood pressure^29^ subjects were instructed to remain seated throughout the entire procedure. SDPP was measured from the beginning to the end of the experiment.

The PPG obtained physiological information by illuminating the skin with a green light-emitting diode and receiving the light with a photodiode (sensitive area 15 mm^2^; 7.5 mm^2^ × 2 sets). The device used the embedded firmware to calculate a blood volume pulse (BVP) signal with reduced noise and motion artifacts. The estimated PP value was calculated as the gain-adjusted ratio of the time to the systolic peak relative to the BVP beat duration. SDPP was calculated using a PPG signal of less than 30 s.

#### HR and HRV

To collect data for HR and HRV analysis, myBeat HR Sensor WHS-3 (Union Tool Co., Tokyo, Japan) was used to record the RR interval (RRI) from the beginning to the end of the experiment. The cardiac cycle mode was selected as the measurement mode, and sampling was performed at a frequency of 1000 Hz. The electrodes of myBeat HR Sensor WHS-3 were placed on the chest 8–10 cm below the clavicle of the participants. The sensor communicated with the dedicated application “WHS-3” on a tablet through Bluetooth, and the measured data were stored on the tablet.

Before calculating HR and HRV, RRI data were preprocessed using the “Toss” Method by Kemper et al.^33^. Specifically, we excluded data that were outside the range of 200–2000 ms (equivalent to 30–300 bpm) and data with a relative difference of RRI of 30% or more from the average value of the last four intervals previously observed. After visually confirming that extreme values and rapid fluctuations in RRI had been removed, the RRI data that had been preprocessed were analyzed using RRI Analyzer 2 (Union Tool Co., Tokyo, Japan). In accordance with the RRI Analyzer 2 specification, filtering was performed to remove RRI data that were outside the range of 250–2000 ms (equivalent to 30–240 bpm). This exclusion criterion is supported by Piskorki et al.^34^. From the preprocessed RRI, HF and LF components were calculated using RRI Analyzer 2, and the index of SNS activity (LF/HF) was calculated by dividing LF by HF.

#### Salivary cortisol

Salivary cortisol levels were measured via enzyme-linked immunosorbent assay using a commercial YK241 cortisol (saliva) EIA kit (Yanaihara Institute Inc.). Saliva was collected into dedicated containers using straws. Sampling was performed three times in the Stressed group: before stress loading (baseline), immediately after stress loading (load), and after a 30-min rest after stress loading (recovery) (see procedures for details). In the Control group, the participants read a book instead of undergoing stress, but sampling was performed at the same time intervals as the intervention group. William et al.^35^reported that cortisol reaches peak levels approximately 38 min after the start of the load phase (or 18 min after its completion). However, other studies, including those by Juster et al. and Dickerson et al., have found that cortisol typically reaches its peak 20 min after the start of the load phase^36,37^ Based on this evidence, the present study adopted this timing for sampling.

#### Self-administered questionnaires

The participants were asked to complete two self-administered psychological evaluations: POMS2 (Kanekoshobo, Tokyo, Japan) and STAI (Sankyobo, Tokyo, Japan). They responded at their own pace with no time limit. These psychological evaluations were also performed three times: baseline, load, and recovery.

POMS2 is a psychological assessment scale used to evaluate fatigue and mood. It has been translated into Japanese, and its reliability and validity have been previously verified^38^. The shortened version of POMS2 consists of 35 items. The participants were asked to rate their mood at that moment on a scale of 0 (not at all) to 4 (very much). POMS2 assesses seven domains: anger–hostility (AH), confusion–bewilderment (CB), depression–dejection (DD), fatigue–inertia (FI), tension–anxiety (TA), vigor–activity (VA), and friendliness (F). The total mood disturbance (TMD) score was calculated as follows:

$${\text{TMD}} = \left( {{\text{AH}} + {\text{CB}} + {\text{ DD}} + {\text{FI}} + {\text{TA}}} \right){-}{\text{VA}}$$

In the analysis, the scores of the seven domains and TMD were used. Higher TMD scores indicate a more negative state of mood.

Anxiety status was measured using STAI^39^. STAI consists of separate self-reported evaluations measuring two distinct anxiety concepts: state anxiety (how the participant is feeling “at the moment”) and trait anxiety (how the participant feels “in general”). We used the widely adopted Japanese version of the STAI, which has been validated in various populations^40^. In this study, only the participants’ emotional state at the moment was relevant; thus, only state anxiety was included in the evaluation. The evaluation consists of 20 statements and is assessed using a 4-point Likert scale. For example, “I feel tense” refers to a question about the participant’s current anxiety. The participants completed the questionnaire in a scale format: (1, Not at all; 2, A little; 3, Somewhat; and 4 Very much so). Higher scores reflect greater anxiety.

### TSST and measurement procedures

**Fig. 1:**
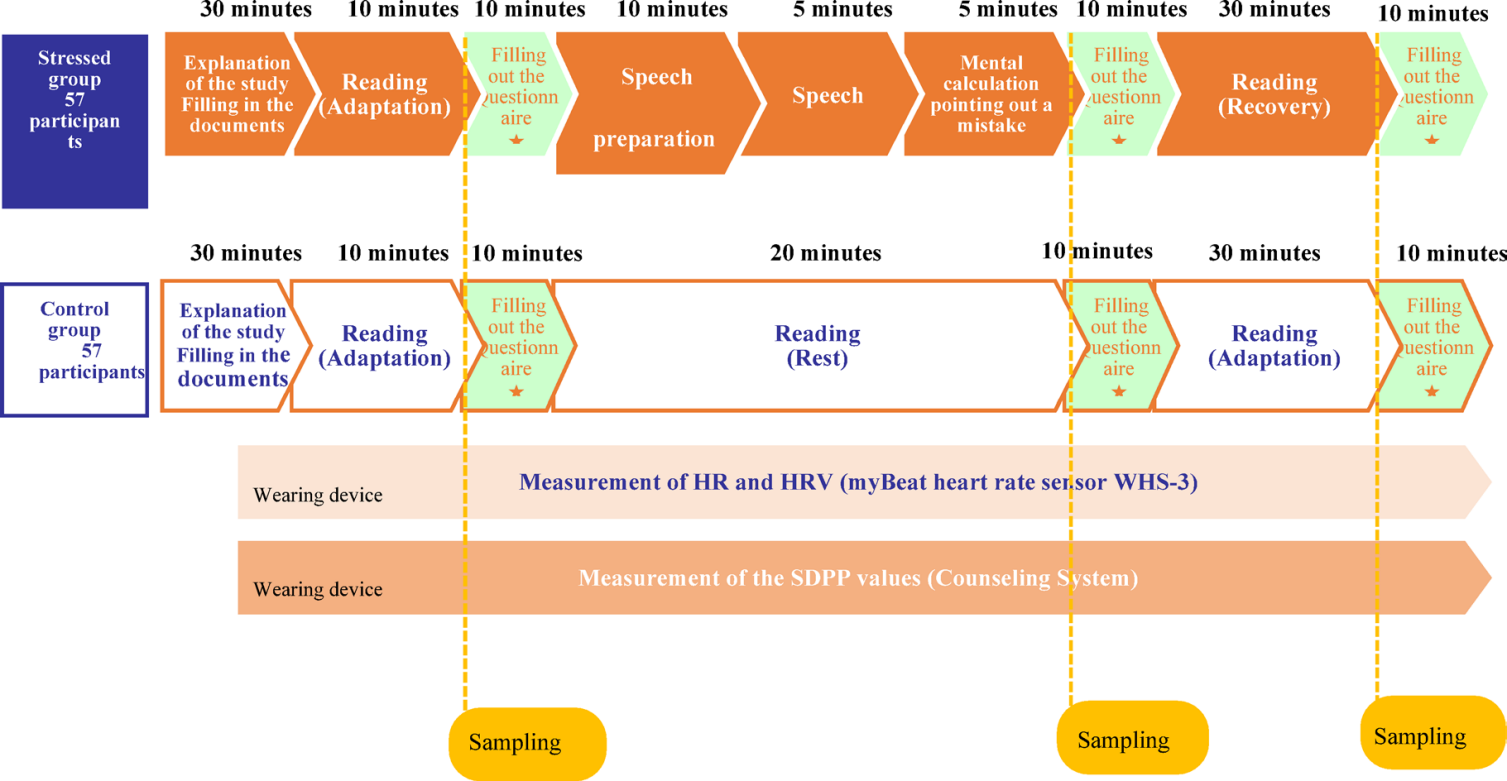
TSST experiment flow.

All the participants received an explanation of the experiment and completed a questionnaire asking for demographic information, such as gender and age. The protocol for the Stressed group included three phases: a 10-min baseline, a 20-min test period (stress test), and a 30-min recovery period (Fig. 1).

#### Baseline

To prepare for saliva collection, the participant rinsed their mouth at the start of the experiment. Then, the Counseling System monitoring device and myBeat Heart Rate Sensor WHS-3 were attached to the participants after being informed that these devices measure pulse waves and HR, respectively. Subsequently, the participants were given a 10-min reading time to help them relax. For reading, we asked the participants to choose from several weekly and monthly magazines containing few or no images to prevent emotional stimulation. After reading, saliva was collected from the participants into dedicated containers using straws. Psychological assessments (POMS2 and STAI) were then conducted.

#### Load (Stress manipulation)

After the psychological evaluations, the Stressed group was given the following explanation regarding the task by the supervisor:

“Now we’re going to explain the tasks. There are two tasks: speech and mental calculation. The speech task is “to introduce yourself well.” Your speech will be 5 min long. Feel free to utilize the notepaper we have provided, but this will be collected before you start your speech. Your speech will be evaluated in real time based on multiple criteria, including the content, your facial expression, and your attitude. You will be videotaped, and evaluators will be taking notes throughout the procedure. Please give your speech facing towards the evaluators and the video camera set up in front of you. We cannot answer any details regarding the evaluation criteria. You will not be allowed to ask any questions during the speech. You are asked to continue talking without interruption of your speech until the time is up. When the time is up, we will let you know. If you have any questions, please ask them now. Now, you will be given 10 min to think about what you are going to talk about.”

The participants prepared for the speech for 10 min, and then provided a verbal speech that lasted for 5 min. At the beginning of the speech, the test supervisor notified the participants, “Now, please start your speech. The content of the speech will be evaluated by the evaluators, and if the speech does not meet the requirements, the time will be extended.” After the speech, the participants performed a mental calculation task (5 min). The instructions from the test supervisor were as follows:

“The next task is subtraction. Subtract 13 from 2097 consecutively. Please answer verbally. Please answer accurately as quickly as possible, If you make a mistake, you will start over from 2097, even if you notice the mistake by yourself. You may now begin.”

The number 2097 was selected based on a protocol implemented at Beppu University in Japan^41^. While the protocols proposed in studies outside Japan (Allen et al. 2014; Kirschbaum et al. 1993; Labuschagne et al. 2019)^18,42,43^ differ in terms of speech preparation time and arithmetic task design, a meta-analysis of TSST studies using cortisol as an indicator found that the differences did not significantly impact cortisol measurements, as indicated by overlapping confidence intervals^44^.

After finishing the speech and mental calculation tasks, the participants collected saliva into dedicated containers using straws. Psychological assessments (POMS2 and STAI) were then conducted.

#### Recovery

After the psychological evaluations, the supervisor instructed the participants as follows: “Please read a magazine for 30 min to relax like in the beginning. However, do not fall asleep.” The participants proceeded to read. Subsequently, they were asked to collect saliva into dedicated containers using straws. Psychological assessments (POMS2 and STAI) were then conducted for the last time before the experiment was completed.

In addition to the supervisor, two additional individuals were recruited as evaluators. During the test period, the participants were asked to perform the procedures in front of the evaluators to induce stress. The evaluators wore lab coats and maintained a neutral expression and composure, and minimized verbal and physical feedback. The evaluators responded only to indicate whether or not to continue the speech, or when there was a mistake during the mental calculation task. To further induce stress in the participants, a video camera and microphone were installed to record the process. The participants were told not to use electronic media during the procedure. SDPP and RRI were continuously measured during the experiment.

Meanwhile, the Control group also had three periods: a 10-min preparation period (baseline), a 20-min reading period (load), and a 30-min recovery period (recovery) (Fig. 1). However, the participants of the Control group were asked to continue reading magazines even during the load period, while the Stressed group was performing speech and mental calculation tasks. SDPP, HR, HF, and LF were continuously measured throughout the experiment. After each of the three periods, the participants collected saliva into dedicated containers using straws. Psychological evaluations (POMS2 and STAI) were then conducted. The experiment was completed after the final psychological evaluation.

### Statistical analysis

For each index, the Mann–Whitney U test was conducted for comparison between the groups across three phases: baseline, load, and recovery. To account for multiple comparisons, the Holm correction method was applied. Within-group comparison across phases for each index was conducted using the Conover test, where the Holm method was again used for multiple comparison correction. Assumptions of normality, homogeneity of variance, and sphericity were violated in some of the indicators, resulting in inconsistent suitability for parametric tests such as ANOVA. To avoid applying statistical tests with different statistical power, which could lead to biased interpretations, nonparametric tests were applied for all indicators. However, it should be noted that differences in the number of missing values across indicators may still result in variations in statistical power.

To determine whether SDPP reflects a stress-induced response that differs from those of HR, LF/HF, HF, and cortisol to better explain the difference between the groups, a binomial logistic regression analysis was conducted. The presence or absence of stress loading served as the response variable (Control group = 0, Stressed group = 1). In the null model, the explanatory variables included HR, LF/HF, HF, and cortisol data, all taken during the load phase, as well as other data including age, sex, and body mass index (BMI). The alternative model additionally included SDPP. Gender was included in both models; because some previous studies have reported gender differences in responses to TSST; for example, men tend to exhibit stronger cortisol responses than women^45–47^.

A multilevel analysis was also conducted to determine whether the addition of SDPP to HR, LF/HF, HF, and cortisol improves the estimation of changes in subjective psychological status through the different phases of the experiment (baseline, load, and recovery) in the Stressed group. The response variables were the TMD score of POMS2, each of the factors in POMS2, and STAI. Level 1 represented the phases (baseline, load, and recovery) within each individual, while Level 2 accounted for differences between individuals. Each explanatory variable was standardized for each individual by subtracting the mean value of the baseline, load, and recovery measurements, then dividing by the standard deviation. The explanatory variables of the null model were HR, LF/HF, HF, and cortisol, whereas the alternative model additionally included SDPP. The analysis was conducted using the random intercept model.

JASP (Amsterdam, The Netherlands) (version 0.18.3^48^) was used for the statistical analyses.

## Results

### Preprocessing for HRV analysis

Although the ECG device used in this study did not permit direct observation of raw ECG waveforms, visual inspection of the exported RRI data revealed numerous abnormal values. These included RRIs at the device’s hardware limits (e.g., > 3000 ms) and abrupt fluctuations, which likely indicate R-wave misdetections due to noise or artifacts in the ECG signal. To address these issues, preprocessing was performed using two filters. First, RRIs outside the physiologically plausible range of 200–2000 ms were excluded. Second, a rolling average filter was applied to remove RRIs that deviated by more than 30% from the mean of the preceding four intervals.

Preprocessing results showed that 19 subjects had more than 1% of their data classified as abnormal. Among these individuals, the mean percentage of abnormal data was 11.8% (SD = 19.1%), with a range of 1.0–74.7%. Following preprocessing, all data were retained for further analysis, as visual inspection confirmed the viability of the remaining RRI data.

### Verification of manipulation with intergroup and inter-phase comparisons of psychological indices

**Fig. 2:**
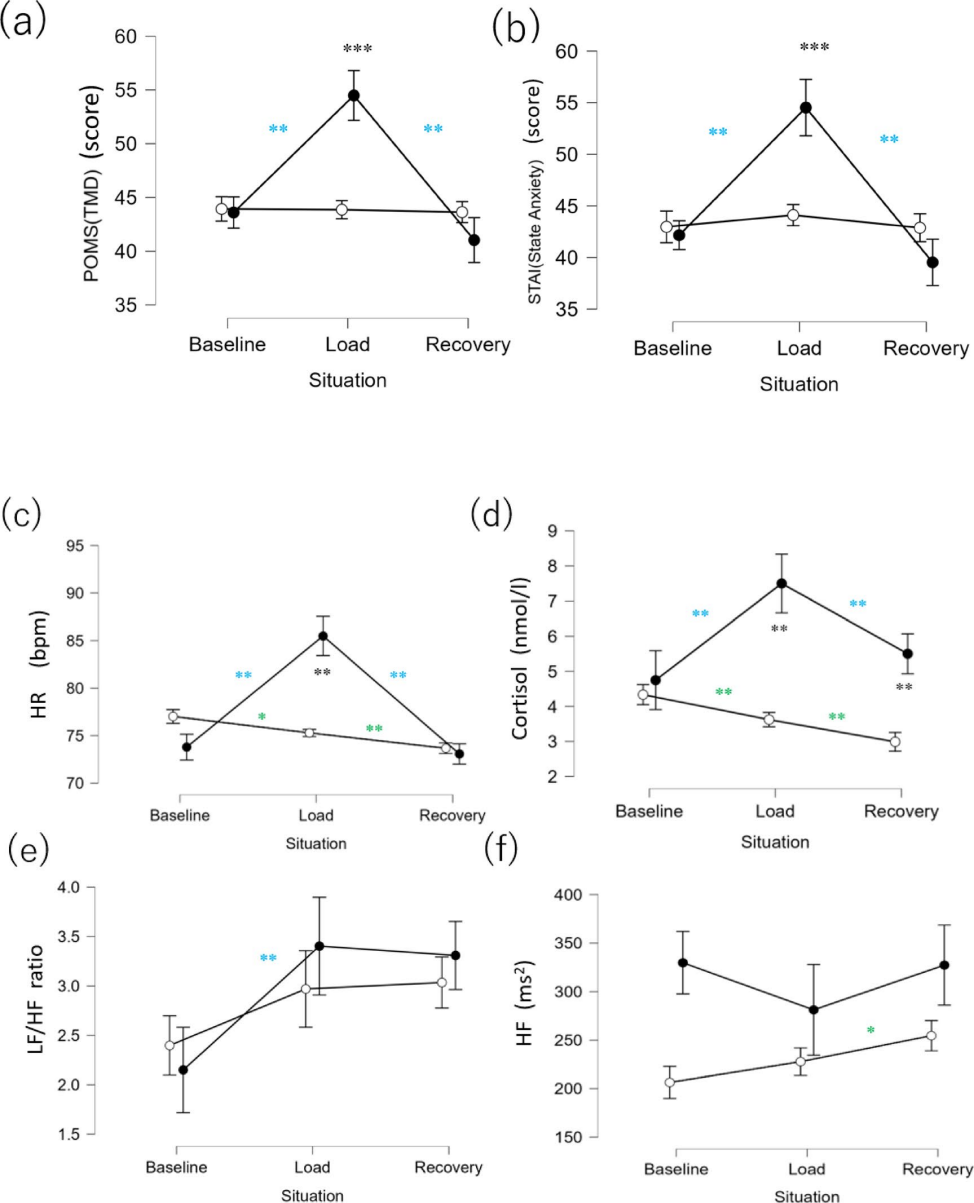

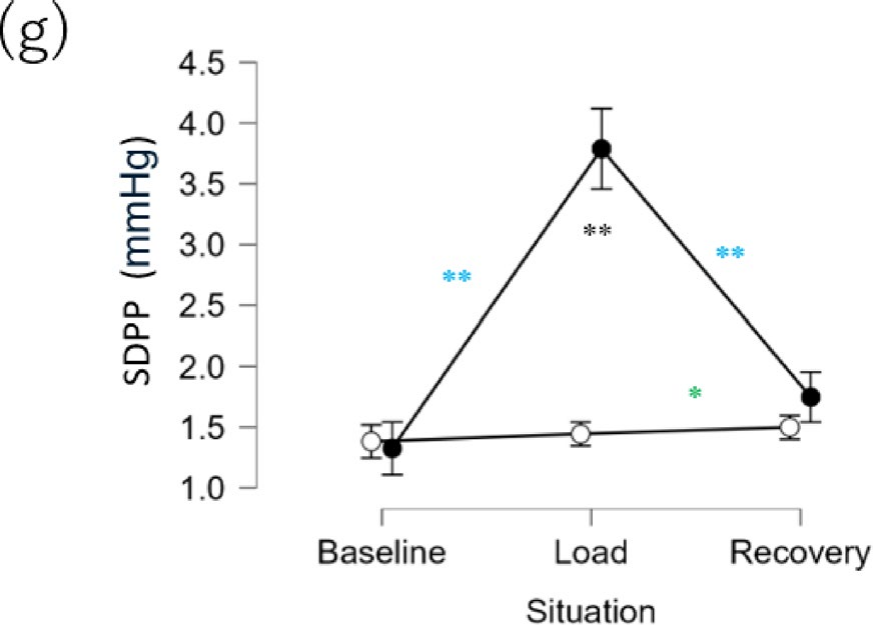
Intergroup and inter-phase comparisons of each item. ● indicates the Stressed group, and **○** indicates the Control group. (**a**) TMD (POMS2) (**b**) State Anxiety (STAI), (**c**) HR, (**d**) cortisol, (**e**) LF/HF, (**f**) HF, (**g**) SDPP. ***P < 0.001, **P < 0.01, *P < 0.05. Comparisons between phases in the Stressed and Control groups are shown in black; those between baseline and load and between load and recovery in the Stressed group are shown in blue; and those between baseline and load and between load and recovery in the Control group are shown in green.

To confirm whether the psychological status had changed due to the stress manipulation in the TSST, we first performed intergroup comparison in each phase for the TMD scores in POMS2 and State Anxiety (STAI). The results indicated that the Stressed group had significantly higher values than the Control group during load (Fig. 2a). No significant difference was observed between the groups in baseline and recovery (Table 1).

**Table 1:** Comparison of statistical results between the stress group and the control group.

Index	Phase	Sample size		*W*	Sig	*r*_*rb*_	95% CI	*W*
		Stress group	Control group					
POMS2(TMD)	Baseline	57	57	1739	–	0.07	− 0.14	0.28
	Load	57	57	784	**	− 0.52	− 0.66	-0.35
	Recovery	57	57	1950	–	0.20	− 0.01	0.39
STAI(State Anxiety)	Baseline	57	57	1641	–	0.01	− 0.20	0.22
	Load	57	57	731	**	− 0.55	− 0.68	− 0.39
	Recovery	57	57	1896	–	0.17	− 0.04	0.36
HR	Baseline	57	55	1782	–	0.14	− 0.08	0.34
	Load	57	55	881	**	− 0.44	− 0.59	− 0.25
	Recovery	57	55	1567	–	0.00	− 0.21	0.21
Cortisol	Baseline	56	57	1574	–	− 0.01	− 0.22	0.20
	Load	57	56	812	**	− 0.49	− 0.64	− 0.31
	Recovery	57	56	825	**	− 0.48	− 0.63	− 0.30
LF/HF	Baseline	53	50	1618	–	0.22	− 0.01	0.40
	Load	54	49	1043	–	− 0.21	− 0.41	− 0.01
	Recovery	54	48	1217	–	− 0.06	− 0.28	0.16
HF	Baseline	54	51	1083	–	− 0.21	− 0.41	0.01
	Load	54	51	1120	–	− 0.13	− 0.34	− 0.09
	Recovery	54	50	1191	–	− 0.19	− 0.33	0.10
SDPP	Baseline	57	57	1579	–	− 0.03	− 0.24	0.18
	Load	57	57	339	**	− 0.79	− 0.86	− 0.70
	Recovery	57	57	1372	–	− 0.16	− 0.35	0.06

The Sig. column indicates significance level symbols. *p* < 0.001, ***p < 0.01, *p < 0.05.

Next, we determined whether there had been a change in psychological status due to the stress manipulation in the Stressed group. The results indicated that in the Stressed group, TMD (POMS2) and State Anxiety (STAI) were significantly higher in load than in baseline (Fig. 2a), but were significantly lower in recovery than in load. Meanwhile, such significant changes were not observed in the Control group (Table 2). See Fig. S1 and Tables S2 and S3 in the Supplemental Information for the results of similar comparison for each factor in POMS2.

**Table 2:** Comparison of statistical results across experimental phases.

Index	Phase	Group	Sample size	*W*	Sig	*r*_*rb*_	95% CI	
POMS2(TMD)	Baseline vs. load	Stress	57 vs. 57	64	**	− 0.92	− 0.96	− 0.86
		Control	57 vs. 57	732	–	0.06	− 0.23	0.35
	Baseline vs. recovery	Stress	57 vs. 57	772	–	0.37	0.09	0.60
		Control	57 vs. 57	710	–	0.03	− 0.26	0.32
	Load vs. recovery	Stress	57 vs. 57	1538	**	0.93	0.87	0.96
		Control	57 vs. 57	606	–	− 0.01	− 0.30	0.28
STAI(State Anxiety)	Baseline vs. load	Stress	57 vs. 57	68	**	− 0.91	− 0.95	− 0.85
		Control	57 vs. 57	556	–	− 0.19	− 0.46	0.10
	Baseline vs. recovery	Stress	57 vs. 57	947	–	0.37	0.09	0.60
		Control	57 vs. 57	767	–	− 0.04	− 0.33	0.25
	Load vs. recovery	Stress	57 vs. 57	1437	**	0.93	0.88	0.96
		Control	57 vs. 57	835	–	0.21	− 0.08	0.47
HR	Baseline vs. load	Stress	57 vs. 57	33	**	− 0.96	− 0.98	− 0.93
		Control	55 vs. 55	1217	*	0.58	0.34	0.75
	Baseline vs. recovery	Stress	57 vs. 57	962	–	0.16	− 0.13	0.43
		Control	55 vs. 55	1398	**	0.82	0.69	0.90
	Load vs.vs. recovery	Stress	57 vs. 57	1648	**	0.99	0.99	1.00
		Control	55 vs. 55	1327	**	0.79	0.64	0.88
Cortisol	Baseline vs. load	Stress	56 vs. 57	213	**	− 0.70	− 0.83	− 0.52
		Control	57 vs. 56	1176	**	0.71	0.52	0.83
	Baseline vs. recovery	Stress	56 vs. 57	585	–	− 0.27	− 0.52	0.03
		Control	57 vs. 56	1356	**	0.83	0.70	0.90
	Load vs. recovery	Stress	57 vs. 57	1370	**	0.66	0.45	0.80
		Control	56 vs. 56	1087	**	0.58	0.34	0.75
LF/HF	Baseline vs. load	Stress	53 vs. 54	213	**	− 0.70	− 0.83	− 0.51
		Control	50 vs. 49	412	–	− 0.33	− 0.58	− 0.02
	Baseline vs. recovery	Stress	53 vs. 54	175	**	− 0.75	− 0.85	− 0.57
		Control	50 vs. 48	246	**	− 0.58	− 0.76	− 0.33
	Load vs. recovery	Stress	54 vs. 54	801	–	0.08	− 0.22	0.37
		Control	49 vs. 48	490	–	− 0.17	− 0.46	0.16
HF	Baseline vs. load	Stress	54 vs. 54	919	–	0.24	− 0.06	0.50
		Control	51 vs. 51	454	–	− 0.29	− 0.55	0.02
	Baseline vs. recovery	Stress	54 vs. 54	702	–	− 0.05	− 0.35	0.25
		Control	51 vs. 50	233	**	− 0.64	− 0.79	− 0.41
	Load vs. recovery	Stress	54 vs. 54	591	–	− 0.20	− 0.47	0.10
		Control	51 vs. 50	306	**	− 0.52	− 0.71	− 0.25
SDPP	Baseline vs. Load	Stress	57 vs. 57	11	**	− 0.99	− 0.99	− 0.98
		Control	57 vs. 57	762	–	− 0.08	− 0.36	0.22
	Baseline vs. recovery	Stress	57 vs. 57	368	**	− 0.55	− 0.73	− 0.32
		Control	57 vs. 57	598	**	− 0.28	− 0.52	0.01
	Load vs. recovery	Stress	57 vs. 57	1608	**	0.95	0.90	0.97
		Control	57 vs. 57	527	*	− 0.36	− 0.59	− 0.08

The Sig. column indicates significance level symbols. *p* < 0.001, ***p < 0.01, *p < 0.05.

These results showed anticipated changes in psychological status due to the stress manipulation in TSST, consistent with previous studies, confirming the validity of the stress intervention.

### Intergroup and inter-phase comparisons of HR, HRV, and cortisol

Comparisons were made between the groups and among phases for conventional physiological indices used for stress. In the intergroup comparisons performed for each phase, HR and cortisol were found to be significantly higher in the Stressed than in the Control group in load (Fig. 2c,d). No significant differences were observed in any of the indices between the groups in baseline (Table 1). In recovery, only cortisol was significantly higher in the Stressed than in the Control group. LF/HF and HF did not exhibit significant differences between the groups (Fig. 2d,e, Table 1).

In the Stressed group, HR and cortisol were higher in load than in baseline, similar to the psychological indices (Fig. 2c,d), and were lower in recovery than in load. LF/HF, an index of HRV, was higher in load than in baseline, and no significant difference was observed between load and recovery (Fig. 2e, Table 1). Moreover, for HF, no significant difference was observed between the three phases (Fig. 2f, Table 1). In the Control group, HR and cortisol decreased over time from baseline to load and recovery, unlike the psychological indices. LF/HF exhibited no significant difference between the phases, whereas HF was higher in recovery than in load (Table 2).

The results indicated that in the Stressed group, HR and cortisol reflected the stress load of TSST and were consistent with the psychological indices, whereas HRV indices, such as LF/HF and HF, showed different patterns. In the Control group, HR and cortisol showed a decrease over time, which was not the case with the psychological indices.

### Intergroup and inter-phase comparisons of SDPP

The main target of this study, SDPP, was similarly compared between the groups and phases. When intergroup comparisons were performed in each phase, similar to the psychological indices and HR, the SDPP was significantly higher in the Stressed group than in the Control group in load (Fig. 2g).

In the Stressed group, HR and cortisol showed higher values in load than in baseline, similar to the psychological indices (Fig. 2g), but lower values in recovery than in load. In the Control group, SDPP increased from load to recovery (Tables 1, 2).

While some of the patterns observed in the Control group were not consistent, SDPP was consistent with the psychological indices, HR, and cortisol, showing that the stress manipulation was reflected by SDPP.

### Logistic regression analysis

Logistic regression analysis was conducted using the measurement values during the load phase to examine whether SDPP can detect stress responses that are different from those reflected by HR, LF/HF, HF, and cortisol. The presence or absence of stress in the two groups served as the response variable. The explanatory variables of the null model (H_0_) were HR, LF/HF, HF, cortisol, age, sex, and BMI, whereas the alternative model (H_1_) additionally included SDPP.

The results of the intermodel comparison confirmed that the H_1_ model with SDPP was superior to the H_0_ model (Table 3: χ^2^(1) = 35.88, *P* < 0.001, McFadden *R*^2^ = 0.344). In addition, as the H_0_ model had AUC = 0.818, sensitivity = 0.704, and specificity = 0.729 while the H_1_ model had AUC = 0.928, sensitivity = 0.87, and specificity = 0.792, a higher degree of explanation accuracy was confirmed for the H_1_ model (Table 3).

**Table 3:** Comparison of logistic regression models using the presence or absence of stress loading as the response variable and the results of estimated coefficients.

	H_o_ model				H_1_ model			
	Estimate		*SE*	Odds ratio	Estimate		*SE*	Odds ratio
Coefficients								
Intercept	− 11.73	***	3.51	8.01 × 10^–6^	− 9.35	*	4.01	8.66 × 10^–5^
SDPP load	–		–		1.58	***	0.38	4.83
HR load	0.08	**	0.03	1.08	0.03		0.03	1.03
LF/HF load	0.12		0.12	1.13	0.07		0.14	1.08
HF load	0.00		0.00	1.00	0.00		0.00	1.00
Cortisol load	0.31	**	0.10	1.37	0.35	**	0.13	1.43
Age	0.06	*	0.03	1.07	0.05		0.04	1.05
Gender	− 0.57		0.53	0.56	− 0.72		0.72	0.49
BMI	0.02		0.07	1.02	− 0.04		0.09	0.96
Model summary								
Deviance	104.45				68.57			
AIC	120.45				86.57			
BIC	141.45				110.19			
*df*	94.00				93.00			
χ^2^	–				35.88			
*p*	–				< 0.001			
McFadden *R*^2^	–				0.34			
Performance								
Accuracy	0.72				0.83			
AUC	0.82				0.93			
Sensitivity	0.70				0.87			
Specificity	0.73				0.79			

The explanatory variables of the null model (H_0_) were HR, LF/HF, HF, cortisol, age, gender, and BMI, whereas those of the alternative model (H_1_) had SDPP in addition to the explanatory variables of the null model.

SE, standard error; AIC, Akaike information criterion; BIC, Bayesian information criterion.

***P < 0.001, **P < 0.01, *P < 0.05. The sample sizes for each were as follows: H_0_ model = 102, H_1_ model = 102.

The estimation results for each explanatory variable using the H_1_ model are presented in Table 3. SDPP and cortisol were the only variables that significantly explained the differences between the groups. The odds ratios indicated that a 1-point increase in SDPP resulted in a 4.83-fold increase in the odds of being in the Stressed group (Table 3). HR significantly explained the difference between the groups in the H_0_ model (Table S4 in the Supplemental Information) but not in the H_1_ model, which had SDPP.

The above are the results of the analysis using data in the load phase, but in the logistic regression analysis using data in the baseline phase, there were no variables that could explain the differences between the groups in either the H_0_ or H_1_ model, which is consistent with the fact that there were no differences between the groups in terms of stress manipulation or in each physiological index (Fig. 2c,d,e,f,g) (Tables S5 and S6 in the Supplemental Information). As cortisol showed a difference between the groups in recovery (Fig. 2d), it significantly explained the difference between the groups in the H_0_ and H_1_ models (Tables S7 and S8 in the Supplemental Information).

These results indicate that with the addition of SDPP to HR, HRV, and cortisol, the difference between the Stressed and Control groups during the load phase can be better explained.

### Multilevel analysis

Through multilevel analysis, we examined whether the addition of SDPP to the explanatory variables improves the estimation of subjective psychological indices (TMD [POMS2] and State Anxiety [STAI]) across different stages within the Stressed group. The response variables were the subjective psychological indices (TMD and State Anxiety). For the explanatory variables, in addition to HR, LF/HF, HF, and cortisol used in the null model (H_0_), the alternative model (H_1_) also had SDPP.

The result of the comparison between the models using the likelihood ratio test confirmed that the H_1_ model was superior for TMD (POMS2) than the H_0_ model (χ^2^(1) = 7.38, *P* = 0.007). Moreover, in H_0_, Akaike information criterion (AIC) and Bayesian information criterion (BIC) values were 1,169 and 1,190, respectively, whereas in H_1_, they were 1164 and 1188, respectively (Table 4). This suggests that the addition of SDPP to the explanatory variables improves the estimation of changes in mood states associated with stress loading (see Tables S11–S17 in the Supplemental Information for the results of each factor in POMS2).

**Table 4:** Comparison of multilevel analysis models using TMD (POMS2) as the response variable and the results of estimated coefficients.

	H_0_ model			H_1_ model		
	Estimate		*SE*	Estimate		*SE*
Coefficients						
Intercept	46.30	***	1.11	46.30	***	1.11
SDPP	–		–	2.86	**	1.04
HR	4.90	***	0.77	2.86	**	1.05
LF/HF	0.71		0.74	0.25		0.73
HF	1.18		0.72	0.46		0.74
Cortisol	1.23		0.72	0.95		0.70
Model summary						
Deviance	1155.37			1147.99		
AIC	1169.37			1163.99		
BIC	1190.72			1188.39		
*df*	7			8		
log Lik	− 577.69			− 574.00		
χ^2^	–			7.38		
*p*	–			0.0066		

The explanatory variables of the null model (H_0_) were HR, LF/HF, HF, and cortisol, whereas those of the alternative model (H_1_) were the explanatory variables of the null model and SDPP. Standardization of the scores (subtracting the mean values of baseline, load, and recovery for each participant from each value, which was then divided by the standard deviation) was performed for explanatory variables. *SE* = standard error; AIC = Akaike information criterion; BIC = Bayesian information criterion; log Lik. = log-likelihood ratio. ***P < 0.001, **P < 0.01, *P < 0.05. The sample sizes for each were as follows: H_0_ model = 52, H_1_ model = 52.

For State Anxiety (STAI), no significant difference was observed between the H_1_ and H_0_ models (H_0_: AIC 1176, BIC 1198; H_1_: AIC 1176, BIC 1201, χ^2^(1) = 2.08, *P* = 0.15) (Table 5). This result does not provide evidence that the addition of SDPP improves the estimation accuracy of STAI. As an exploratory analysis, the effect of SDPP’s addition was examined after excluding the HRV indices, as its inclusion considerably reduced the available sample size due to the high occurrence of abnormal HRV values, and also because the HRV results were not consistent with the trends from past studies. The results confirmed that the H_1_ model with SDPP was superior to the H_0_ model (χ^2^(1) = 6.48, *P* = 0.01). In H_0_, the AIC and BIC were 1263 and 1278, respectively, whereas in H_1_, they were 1258 and 1277, respectively (see Table S18 in the Supplemental Information for details of the results). This indicated that for State Anxiety, adding SDPP to the response variable improves the estimation accuracy, when HRV indices are not used.

**Table 5:** Comparison of multilevel analysis models using State Anxiety (STAI) as the response variable and the results of the estimated coefficients.

	H_0_ model			H_1_ model		
	Estimate		*SE*	Estimate		*SE*
Coefficients						
Intercept	45.58	***	1.03	45.58	***	1.03
SDPP	–		–	1.65		1.14
HR	5.83	***	0.83	4.66	***	1.16
LF/HF	0.68		0.80	0.41		0.81
HF	0.87		0.77	0.45		0.82
Cortisol	0.90		0.77	0.74		0.77
Model summary						
Deviance	1162.32			1160.25		
AIC	1176.32			1176.25		
BIC	1197.67			1200.64		
*df*	7			8		
log Lik	− 581.16			− 580.12		
χ^2^	–			2.08		
*p*	–			0.1494		

The explanatory variables of the null model (H_0_) were HR, LF/HF, HF, and cortisol, whereas those of the alternative model (H_1_) were the explanatory variables of the null model and SDPP. Standardization of the scores (subtracting the mean values of baseline, load, and recovery for each participant from each value, which was then divided by the standard deviation) was performed for the explanatory variables.

SE, standard error; AIC, Akaike information criterion; BIC, Bayesian information criterion; log Lik., log-likelihood ratio.

***P < 0.001, **P < 0.01, *P < 0.05. The sample sizes for each were as follows: H_0_ model = 52, H_1_ model = 52.

In summary, these results indicate that the addition of SDPP improved the accuracy of estimating mood state changes associated with stress loading in the Stressed group, as shown with TMD (POMS2). For State Anxiety, the findings indicate that SDPP contributes to improved estimation accuracy when HRV indices are not used.

## Discussion

### Comparison of the stressed group and the control group by the presence or absence of stress load

We investigated whether SDPP reflects stress, and whether adding SDPP to conventional indices can improve stress estimation. In this study, TSST was used to induce psychological stress. The differences in the phases (baseline, load, and recovery) between the Stressed group and the Control group were examined to evaluate the effectiveness of the stress manipulation. During the load period of the Stressed group, unlike the Control group, there was an increase in physiological and psychological indices, such as HR and cortisol (Fig. 2a,b,c,d). Trends of HR and cortisol were consistent with those of previous TSST studies^44,49–51^, suggesting that stress was successfully induced. However, this was not the case for the HRV indices, HF and LF/HF. Possible reasons include body movement, improper electrode placement (which may have introduced myoelectric noise), and weak input signal. These factors could have led to incorrect R-wave detection, resulting in abnormal RRI data. Frequency-domain indices such as HF are sensitive to RRI inaccuracies, as they affect the construction of the evenly spaced time series required for spectral analysis^52^. Moreover, the temporal structure of the signal may have been distorted by the removal of abnormal RRIs during filtering, potentially affecting the results of the frequency domain analysis. In contrast, HR, although calculated from the same 2-min data window, appears to be less affected, as it is derived from a simple average of RRI within each window^53^.

For SDPP, the increase in SDPP from baseline to load was observed only in the Stressed group (Fig. 2g). These results suggest that SDPP is capable of detecting stress, similar to conventional stress indices such as HR and cortisol. Although the underlying mechanism is currently unknown, it is hypothesized that SDPP is influenced by SNS activation, similar to the other indices such as HR and LF/HF. Its heightened sensitivity to stress over traditional HRV indices may be attributed to its nature as a time-domain measure, like HR, and its dependence on shorter time segments (< 30 s). This approach likely reduces susceptibility to noise and temporal distortions, potentially making SDPP more robust than frequency-domain HRV indices in noisy environments. Other studies have established that an increase in the indices that are derived from changes in SBP or the standard deviation of mean blood pressure indicates an activation of the SNS^54–58^. It has also been reported^59^ that SDPP, may represent blood pressure fluctuations better than merely looking at the variability of SBP^60^, as it reflects left ventricular ejection fraction, early pulse wave reduction, and pulse rate. Yang, Kuo et al. further reported through rat experiments, that respiration-related arterial pressure fluctuations may be promoted by SNS function, which can be reflected in the PP variability^61^. These reports support the theory that SDPP reflects SNS activity.

### SDPP as a distinct indicator of stress response: findings from logistic and multilevel analyses

Logistic regression and multilevel analyses were conducted to determine whether SDPP reflects stress responses not reflected by conventional stress indices (HR, LF/HF, HF, and cortisol). The results of the logistic regression analysis, using the difference between groups (i.e., presence or absence of stress load) as the response variable, indicated that during the load phase, the difference between the Stressed group and the Control group could be better explained by including SDPP in the explanatory variables. Moreover, a multilevel analysis using TMD (POMS2) and State Anxiety (STAI) as the response variables in the Stressed group revealed that the addition of SDPP allows for improved explanatory power of the changes in TMD (POMS2) within Level 1 (different phases of baseline, load, and recovery). For STAI, when explorative analyses were conducted excluding the HRV indices (LF/HF and HF), due to reduced sample size and inconsistent trends mentioned earlier, the estimation accuracy was increased by the addition of SDPP to the explanatory variables. Taken together, these results suggest that SDPP reflects stress responses different from those reflected by conventional physiological stress indices, allowing for better estimation of stress when used in combination with other indices.

SDPP is considered a SAM-related response and is thought to reflect a stress response that is distinct from that of cortisol, which is associated with the HPA axis.

Furthermore, SDPP captures aspects of stress responses unique from HR and HRV because PP is influenced not only by cardiac output but also by total peripheral vascular resistance (TPR). The following physiological mechanisms are hypothesized: Acute stress activates the SNS, leading to β1-adrenergic stimulation and α1-adrenergic stimulation, which increases the cardiac output and TPR, respectively^62^. An increase in TPR, reflecting a decrease in vascular compliance, results in elevated SBP. Rosa et al. demonstrated that intravenous administration of an α1-adrenergic receptor-selective inhibitor resulted in no change in cardiac output, while TPR and the standard deviation of SBP decreased significantly^63^. On the other hand, reduced vascular compliance steepens the pressure–volume curve, leading to more rapid fluctuations in DBP^64^, which may contribute to differences in DBP and SBP behavior^65^. This suggests that SNS activation may lead to greater SBP variability, thereby increasing SDPP. Furthermore, elevated cardiac output is associated with increased beat-to-beat variability^63^, contributing to SBP fluctuations and higher SDPP. Girard et al. reported that administration of a β1-adrenergic receptor selective antagonist to hypertensive patients significantly reduced the standard deviation of SBP and HR^66^. Individuals experiencing autonomic nervous system activation due to mental stress exhibit similar attenuation of blood pressure response as that observed with β1-adrenergic receptor blockade. These findings suggest that both α1-adrenergic stimulation and β1-adrenergic stimulation contribute to SDPP elevation.

James et al. observed a “mixed” response pattern, where the changes in TPR and cardiac output exhibited a synergistic relationship, potentially enhancing the explanatory power of SDPP^67^.

Based on these findings, SDPP, while dependent on SNS activity like HR and HRV, captures different aspects of the stress response by reflecting changes in circulatory dynamics such as TPR. Specifically, it is suggested that changes in cardiac output and TPR synergistically influence SDPP, giving it significance as a physiological indicator different from both HRV and HPA system indicators. Therefore, utilizing SDPP in combination with heart rate-related indicators like HR and HRV, as well as HPA axis indicators such as cortisol that differ from these SAM system indicators, is thought to enable a more comprehensive evaluation of stress responses.

### Differences between the results of logistic and multilevel analyses

For certain indices, results from the logistic regression and multilevel analysis showed different trends. In the logistic regression analysis, HR’s explanatory power decreased significantly in the H_1_ model, which had the addition of SDPP. The high explanatory power of SDPP may be attributed to the two types of changes associated with stress loading mentioned earlier; an increase in cardiac output, which is closely related to HR, and an increase in TPR. The lower explanatory power of HR could be attributed to the significant reduction in HR from load to recovery in both the Control and Stressed groups. This significant decrease in the Control group may be attributed to the cognitive load during the initial phases of the experiment, which may have induced stress onto the participants, leading to an increased cardiac activity and a heightened HR before the recovery phase, even for the Control group.

Meanwhile, in the multilevel analysis for the scores in POMS2 and STAI (Supplemental Information, Tables S11 to S17), HR’s explanatory power was retained in the H_1_ model, for example, for TMD (POMS2). This may be due to the fact that the Control group’s data was excluded from the multilevel analysis, so the effect of the cognitive load at the beginning of the experiment discussed earlier would not have been as prominent. Another explanation could be that certain subjective psychological stress responses are more correlated with HR, which is calculated solely by cardiac output, than with SDPP, which is influenced by both cardiac output and TPR.

Cortisol also showed different trends between the logistic regression and multilevel analysis. Though cortisol was a significant variable explaining the difference between the groups in the logistic regression analysis, it could not explain TMD (POMS2) as the response variable in the multilevel analysis. As mentioned previously, cortisol is known to have a delayed response, but this did not affect the logistic regression analysis, as the baseline data was compared to load. However, the delayed response becomes more pronounced in the recovery period, as the cortisol levels fail to recover to the baseline state. This could explain the reduced explanatory power of cortisol in the multilevel analysis, which included data from the recovery period. Similar trends were observed in the multilevel analysis using each factor of POMS2 and STAI as the response variable, supporting this theory.

### Differential contributions of SDPP and HR to the estimation of stress-related emotions

As mentioned earlier, differences were observed in the explanatory power of HR and SDPP between the two psychological assessments, POMS2 and STAI. While both SDPP and HR had significant explanatory power for the multilevel analysis of TMD (POMS2), HR had higher explanatory power than SDPP for STAI. This could be explained by differences in the scope of these two evaluations. TMD (POMS2) includes questions regarding emotions such as depression, as well as feelings including guilt, shame, aversion, and anger. On the other hand, STAI is not as comprehensive, as it does not cover certain negative emotions apart from anxiety and depression, such as feelings of guilt, shame, aversion, and anger^68^. Anxiety and depression evaluated by STAI most likely reflect dimensions of stress associated with increased cardiac output compared with TMD (POMS2), which would explain the higher explanatory power of HR.

In the multilevel analysis of STAI, SDPP became a significant explanatory variable only when LF/HF and HF were removed. Because the inclusion of HRV resulted in reduced sample size due to the presence of abnormal data that had to be omitted, this is thought to have affected the explanatory power of SDPP for STAI (Table S19 in the Supplemental Information). This would explain the increased explanatory power of SDPP when HRV was excluded, which resulted in an increased sample size.

It was found that the addition of SDPP improved the estimation in emotions, including depression, anxiety, guilt, shame, and aversion, obtained from TMD (POMS2). Moreover, SDPP slightly improved the estimation for STAI, except when HRV indices were included (Table S18 in the Supplemental Information). When capturing SNS activity or evaluating stress responses, it is necessary to choose an index that reflects the specific type of stress-related emotion being assessed. These findings suggest that SDPP reflects physiological responses associated with emotional changes.

### Limitations

This study has several limitations that need to be acknowledged. Firstly, the HRV response to stress observed in this study was not consistent with results from previous studies, most likely due to noise causing misdetection in R waves. With cleaner HRV data, the relative contribution of HRV indices may increase in logistic analysis and multilevel analysis, potentially reducing the observed superiority of SDPP. This study focused on stress responses measurable by wearable devices, which may have contributed to the signal noise. To better examine the relationship between SDPP and HRV, alternative approaches should be considered for more robust HRV data, such as using devices that are less prone to noise artifacts. It should also be acknowledged that while SDPP’s simplicity offers an advantage in handling noise-prone data often found in wearable devices, the lack of spectral resolution limits is ability to differentiate sympathetic and parasympathetic activity. The relative advantages and limitations in handling noisy data also require further investigation, as inducing noise was not the focus of this study, given that PPG-derived signals are sensitive to factors such as body movement and skin contact.

Secondly, as discussed earlier, HR and cortisol were higher in baseline than in the load and recovery phases of the Control group. This could be attributed to the participants becoming more psychologically stable and less tense over the course of the TSST, resulting in decreased HR. This could have led to suppression of the autonomic nervous system and the endocrine system, causing the decrease in cortisol levels. A previous study suggests that factors such as the mode of transportation used by the participants to come to the testing area and anxiety from interacting with strangers (such as supervisors of the experiment) may have influenced stress and the SNS activation^69^. It has also been reported that it takes up to 60 min for the body to return to its default physiological state after physical activity^70^. In this study, baseline measurements were taken approximately 40 min after the participants entered the laboratory, which may not have allowed sufficient time for the HR and cortisol to normalize. This suggests that the timing of the baseline phase may not have been optimal. In future studies, it would be desirable to provide sufficient time before starting the experiment so that the baseline phase can start after the subject has recovered to the default physiological state. Additionally, William et al. has reported that cortisol reach peak levels approximately 38 min after the start of the load phase (or 18 min after its completion)^46^, but in this study, samples were collected immediately after the TSST ended, potentially missing the peak response. However, William et al. has also reported that across 186 studies, the peak timings were inconsistent, but the general trend of gradual increase and decrease in cortisol levels associated with endocrine dynamics were consistently observed throughout the load and recovery phases. Despite the potential missed peaks, cortisol levels were significantly higher in load than in recovery, indicating that the stress response in TSST was adequately captured.

Thirdly, SDPP has been identified as a stress indicator distinct from HR and cortisol; however, further investigation is needed to understand its specificity. For instance, if SDPP is sensitive to SNS activation, it could serve as an indicator of milder forms of mental load that are not considered as psychological stress. Psychological stress arises in situations involving emotional components, such as threats or social evaluation, and is often accompanied by activation of the HPA axis and the SNS. In contrast, mental load stemming from high cognitive demands associated with task performance does not necessarily involve emotional responses, and triggers strong activation of the SNS only. Wiemers et al. conducted^71^a friendly-TSST to manipulate mental load. This version is designed to provide participants with a stress-free environment through friendly interactions with experimenters, thereby avoiding activation of the HPA axis. Wiemers et al. found that while friendly-TSST did not affect the cortisol levels (indicating minimal activation of HPA axis), α-amylase levels (associated with SAM) increased. This suggests that even in the friendly-TSST, some form of mental loads associated with the tasks and the social situation persist, leading to the activation of the SNS. In future studies, measuring SDPP during the friendly-TSST may help determine the specificity of SDPP to SNS responses under mental load. If SDPP is sensitive to changes in SNS activity, it may capture SNS activation due to cognitive load even with weak emotional stress responses. This could make it a useful measurement tool for depression and non-emotional mental states, that are associated with minimal emotional components.

Fourthly, to ensure participant comfort and standardized measurements, this study had the subjects remain seated throughout the TSST. Because the standard TSST protocol typically involves standing, only a few studies directly compare physiological responses (e.g. blood pressure, autonomic indicators, and hormonal levels) between seated and standing positions during the TSST. While existing literature documents posture information, it lacks statistical comparisons of its effects. Mlynarik et al. investigated the impact of posture change by asking healthy adult males to transition from a seated to a standing position^72^. This resulted in mild increases in systolic and diastolic blood pressure, heart rate, plasma norepinephrine, renin activity, and aldosterone. This indicates that posture changes primarily induce minor responses in the cardiovascular system. In this study, the consistent use of the seated posture minimized variability associated with postural changes, allowing for a more direct evaluation of the responses from the psychological stress. However, since this study did not compare the seated condition with a standing condition, the results reflect stress responses exclusively under seated conditions, which may affect the generalizability of the findings. Future research should directly compare seated and standing conditions to clarify their effects.

Finally, to effectively utilize SDPP in clinical settings for mental health, it is necessary to assess its ability to capture acute stress, SNS activation, and anxiety. For instance, investigating SDPP’s ability to detect changes in the severity of stress responses and to monitor progress towards recovery may help determine its usefulness in exposure therapy for clients with PTSD. However, certain clients with depression or other conditions may have reduced stress-induced activation of SNS. In such cases, it may be difficult to observe physiological reactions using SDPP. Furthermore, when applying SDPP in real clinical settings, several technical challenges must be considered. For instance, data quality of continuous pulse wave measurement using wearable devices may be affected by wearing conditions and other factors such as such as movement and external noise. To ensure reliable continuous measurements, considerations must be made on improvements in both software and device hardware components, including usability and wearability enhancements, for minimizing burden during prolonged use. Furthermore, SDPP was measured using a newly developed non-medical wearable device for this study. Before SDPP can be used in clinical applications, the validity of SDPP measurements using this wearable device must be further verified through comparison with SDPP measurement results obtained using existing medical devices.

To enhance the utility of SDPP in the future, it should be integrated with emerging technologies that enable multifaceted stress assessment, rather than relying on SDPP alone. For instance, combining SDPP with AI technologies, such as facial expression recognition, voice analysis, and text analysis, could potentially help capture types of stress that SDPP struggles to capture, including internal stress, which has subtle effects on the SNS^73–75^. Building such a comprehensive evaluation system is expected to further enhance SDPP’s clinical utility in assessing psychological stress.

## Conclusion

In this study, TSST was conducted in healthy participants to determine whether SDPP, measured using a wearable device, can serve as an index for stress assessment and improve the accuracy of estimating acute psychological stress. The results show that SDPP can be used to detect stress responses, similar to conventional objective stress indices (e.g., HR, cortisol) and subjective indices (i.e., psychological assessments). Moreover, adding SDPP to other indices improved the accuracy of estimating the presence of stress and stress-related emotions as evaluated through subjective psychological assessments. In modern society, the abundance of physical and mental triggers makes psychological stress unavoidable, contributing to various health issues. SDPP may be a valuable index for improving the estimation of acute stress and its associated emotions, aiding in the prevention and management of stress-related health issues.

## Supplementary Information

Below is the link to the electronic supplementary material.

Supplementary Material 1

## Data Availability

The source data for the graphs and charts in the figures are available as Supplementary information files. The data that support the graphs within this paper and other findings of this study are available from the corresponding author upon reasonable request.
