# Supplementary material for: Standard deviation of pulse pressure measured using wearable devices improves the estimation of acute psychological stress
Source: Sci Rep. 2025 Nov 20;15:40964. doi: 10.1038/s41598-025-24704-2 (PMC12635272; doi:10.1038/s41598-025-24704-2)
Supplement: Supplementary file 1 — Supplementary Material 1 [file 41598_2025_24704_MOESM1_ESM.pdf]

Supplementary information

Table S-1. Demography of Participants

	Control group	Stress group
Sample size	57	57
Age, mean ± *SD*	44.5±9.6	44.9±11.0
Body height, mean ± *SD*	166.5±8.7	163.8±9.7
Weight, mean ± *SD*	60.5±14.2	59.5±13.3
Sex, n (%)		
Men	28(49)	28(49)
Women	29(51)	29(51)
Health status, n (%)		
Good	32(56)	33(58)
Normal	24(42)	22(39)
Bad	1(2)	2(3)
Medication taking, n (%)		
Yes	3(5)	4(7)
None	54(95)	53(93)
Pre-existing disease, n (%)		
Yes	2(4)	2(4)
None	55(96)	55(96)
Smoking status, n (%)		
Yes	2(4)	5(9)
None	55(96)	52(91)

Table S-2 Comparison between Stress Group and Control Group in Each Situation

Index	Situation	Sample size		W	p value	r_{rb}	95% CI	
		Stress group	Control group					
SDPP	Baseline	57	57	1579	0.799	-0.03	-0.24	0.18
	Load	57	57	339	0.003	-0.79	-0.86	-0.70
	Recovery	57	57	1372	0.306	-0.16	-0.35	0.06
HR	Baseline	57	55	1782	0.426	0.14	-0.08	0.34
	Load	57	55	881	0.003	-0.44	-0.59	-0.25
	Recovery	57	55	1567	1.000	0.00	-0.21	0.21
LF/HF	Baseline	53	50	1618	0.162	0.22	-0.01	0.40
	Load	54	49	1043	0.162	-0.21	-0.41	-0.01
	Recovery	54	48	1217	0.599	-0.06	-0.28	0.16
HF	Baseline	54	51	1083	0.180	-0.21	-0.41	0.01
	Load	54	51	1120	0.512	-0.13	-0.34	-0.09
	Recovery	54	50	1191	0.512	-0.19	-0.33	0.10
Cortisol	Baseline	56	57	1574	0.902	-0.01	-0.22	0.20
	Load	57	56	812	0.002	-0.49	-0.64	-0.31
	Recovery	57	56	825	0.002	-0.48	-0.63	-0.30
POMS2(AH)	Baseline	57	57	1479	0.404	-0.09	-0.29	0.12
	Load	57	57	1297	0.186	-0.20	-0.40	0.01
	Recovery	57	57	1912	0.198	0.18	-0.03	0.37
POMS2(CB)	Baseline	57	57	1722	1.000	0.06	-0.15	0.27
	Load	57	57	472	0.003	-0.71	-0.80	-0.59

	Recovery	57	57	1723	1.000	0.06	-0.15	0.27
POMS2(DD)	Baseline	57	57	1606	0.916	-0.01	-0.22	0.20
	Load	57	57	1135	0.015	-0.30	-0.48	-0.10
	Recovery	57	57	1804	0.606	0.11	-0.10	0.31
POMS2(FI)	Baseline	57	57	1736	0.528	0.07	-0.14	0.27
	Load	57	57	924	0.003	-0.43	-0.59	-0.24
	Recovery	57	57	1907	0.218	0.17	-0.04	0.37
POMS2(TA)	Baseline	57	57	1658	0.851	0.02	-0.19	0.23
	Load	57	57	576	0.003	-0.65	-0.75	-0.50
	Recovery	57	57	1984	0.078	0.22	0.01	0.41
POMS2(VA)	Baseline	57	57	1456	0.680	-0.10	-0.31	0.11
	Load	57	57	1487	0.680	-0.09	-0.29	-0.13
	Recovery	57	57	1407	0.651	-0.13	-0.34	0.08
POMS2(F)	Baseline	57	57	1635	1.000	0.01	-0.20	0.22
	Load	57	57	1729	1.000	0.06	-0.15	0.27
	Recovery	57	57	1693	1.000	0.04	-0.17	0.25
POMS2(TMD)	Baseline	57	57	1739	0.519	0.07	-0.14	0.28
	Load	57	57	784	0.003	-0.52	-0.66	-0.35
	Recovery	57	57	1950	0.132	0.20	-0.01	0.39
STAI(State Anxiety)	Baseline	57	57	1641	0.930	0.01	-0.20	0.22
	Load	57	57	731	0.003	-0.55	-0.68	-0.39
	Recovery	57	57	1896	0.248	0.17	-0.04	0.36

Note: SDPP, Standard deviation of pulse pressure; HR, heart rate; HF, high frequency component of heart rate variability; LF: low frequency component of heart rate variability; POMS2: psychological assessment scale used to evaluate fatigue and mood status. The POMS2 assesses seven domains: Anger-Hostility (AH), Confusion-Bewilderment (CB), Depression-Dejection (DD), Fatigue-Inertia (FI), Tension-Anxiety (TA), Vigour-Activity (VA), and Friendliness (F), and the Total Mood Disturbance (TMD) score is calculated using the formula: TMD = (AH + CB + DD + FI + TA) - VA. STAI (State Anxiety): a separate self-reported rating scale that measures the anxiety concept of state anxiety (how the participant is feeling "at this moment"). r_{rb} rank-biserial correlation coefficient. 95%CI: 95% confidence interval (CI) of r_{rb}. Mann-Whitney U-tests were used for comparison between groups for each of Situation (Baseline), Situation(Load) and Situation (Recovery), and multiple comparison correction using the Holm method was performed to compare these three situations.

Table S3 Comparison in situations in each group (Stress group and Control group)

Index	situation	Group	Sample size	W	p value	r_{rb}	95% CI,	
SDPP	Baseline vs Load	Stress	57 vs 57	11	0.002	-0.99	-0.99	-0.98
		Control	57 vs 57	762	0.926	-0.08	-0.36	0.22
	Baseline vs Recovery	Stress	57 vs 57	368	0.006	-0.55	-0.73	-0.32
		Control	57 vs 57	598	0.008	-0.28	-0.52	0.01
	Load vs Recovery	Stress	57 vs 57	1608	0.002	0.95	0.90	0.97
		Control	57 vs 57	527	0.010	-0.36	-0.59	-0.08
HR	Baseline vs Load	Stress	57 vs 57	33	0.002	-0.96	-0.98	-0.93
		Control	55 vs 55	1217	0.013	0.58	0.34	0.75
	Baseline vs Recovery	Stress	57 vs 57	962	0.542	0.16	-0.13	0.43
		Control	55 vs 55	1398	0.002	0.82	0.69	0.90
	Load vs Recovery	Stress	57 vs 57	1648	0.001	0.99	0.99	1.00
		Control	55 vs 55	1327	0.001	0.79	0.64	0.88
LF/HF	Baseline vs Load	Stress	53 vs 54	213	0.002	-0.70	-0.83	-0.51
		Control	50 vs 49	412	0.156	-0.33	-0.58	-0.02
	Baseline vs Recovery	Stress	53 vs 54	175	0.002	-0.75	-0.85	-0.57
		Control	50 vs 48	246	0.002	-0.58	-0.76	-0.33
	Load vs Recovery	Stress	54 vs 54	801	0.634	0.08	-0.22	0.37
		Control	49 vs 48	490	0.172	-0.17	-0.46	0.16

HF	Baseline vs Load	Stress	54 vs 54	919	0.154	0.24	-0.06	0.50
		Control	51 vs 51	454	0.154	-0.29	-0.55	0.02
	Baseline vs Recovery	Stress	54 vs 54	702	0.731	-0.05	-0.35	0.25
		Control	51 vs 50	233	0.002	-0.64	-0.79	-0.41
	Load vs Recovery	Stress	54 vs 54	591	0.194	-0.20	-0.47	0.10
		Control	51 vs 50	306	0.002	-0.52	-0.71	-0.25
Cortisol	Baseline vs Load	Stress	56 vs 57	213	0.002	-0.70	-0.83	-0.52
		Control	57 vs 56	1176	0.002	0.71	0.52	0.83
	Baseline vs Recovery	Stress	56 vs 57	585	0.202	-0.27	-0.52	0.03
		Control	57 vs 56	1356	0.002	0.83	0.70	0.90
	Load vs Recovery	Stress	57 vs 57	1370	0.002	0.66	0.45	0.80
		Control	56 vs 56	1087	0.007	0.58	0.34	0.75
POMS2(AH)	Baseline vs Load	Stress	57 vs 57	256	0.152	-0.41	-0.62	-0.13
		Control	57 vs 57	309	0.380	0.10	-0.19	0.38
	Baseline vs Recovery	Stress	57 vs 57	353	0.010	0.62	0.40	0.77
		Control	57 vs 57	307	0.660	0.16	-0.13	0.43
	Load vs Recovery	Stress	57 vs 57	620	0.002	0.86	0.76	0.92
		Control	57 vs 57	172	0.660	-0.02	-0.31	0.27
POMS2(CB)	Baseline vs Load	Stress	57 vs 57	4	0.002	-0.99	-1.00	-0.99
		Control	57 vs 57	539	0.041	0.38	0.10	0.60

	Baseline vs Recovery	Stress	57 vs 57	371	0.248	0.41	0.13	0.62
		Control	57 vs 57	642	0.030	0.30	0.01	0.54
	Load vs Recovery	Stress	57 vs 57	1525	0.002	0.98	0.96	0.99
		Control	57 vs 57	202	0.696	-0.07	-0.36	0.22
POMS2(DD)	Baseline vs Load	Stress	57 vs 57	181	0.002	-0.60	-0.76	-0.37
		Control	57 vs 57	374	0.926	0.33	0.05	0.57
	Baseline vs Recovery	Stress	57 vs 57	427	0.006	0.62	0.40	0.77
		Control	57 vs 57	395	0.008	0.50	0.24	0.69
	Load vs Recovery	Stress	57 vs 57	759	0.002	0.94	0.90	0.97
		Control	57 vs 57	266	0.010	0.22	-0.07	0.48
POMS2(FI)	Baseline Vs Load	Stress	57 vs 57	92	0.002	-0.87	-0.92	-0.77
		Control	57 vs 57	450	0.959	-0.17	-0.44	0.13
	Baseline vs Recovery	Stress	57 vs 57	375	0.162	-0.13	-0.40	0.17
		Control	57 vs 57	338	0.144	-0.37	-0.60	-0.10
	Load vs Recovery	Stress	57 vs 57	1191	0.002	0.87	0.77	0.93
		Control	57 vs 57	246	0.080	-0.43	-0.64	-0.16
POMS2(TA)	Baseline vs Load	Stress	57 vs 57	67	0.002	-0.91	-0.95	-0.84
		Control	57 vs 57	830	0.006	0.47	0.21	0.67
	Baseline vs Recovery	Stress	57 vs 57	792	0.001	0.84	0.73	0.91
		Control	57 vs 57	898	0.001	0.66	0.46	0.80

	Load vs Recovery	Stress	57 vs 57	1480	0.002	0.99	0.99	1.00
		Control	57 vs 57	336	0.182	0.45	0.18	0.65
POMS2(VA)	Baseline vs Load	Stress	57 vs 57	1028	0.005	0.49	0.24	0.68
		Control	57 vs 57	671	0.002	0.64	0.43	0.78
	Baseline vs Recovery	Stress	57 vs 57	630	0.045	0.27	-0.02	0.52
		Control	57 vs 57	639	0.004	0.41	0.14	0.63
	Load vs Recovery	Stress	57 vs 57	407	0.824	-0.25	-0.50	0.05
		Control	57 vs 57	286	0.824	-0.04	-0.32	0.25
POMS2(F)	Baseline vs Load	Stress	57 vs 57	1005	0.002	0.64	0.43	0.78
		Control	57 vs 57	916	0.003	0.62	0.41	0.77
	Baseline vs Recovery	Stress	57 vs 57	650	0.010	0.51	0.26	0.70
		Control	57 vs 57	829	0.019	0.53	0.29	0.71
	Load vs Recovery	Stress	57 vs 57	280	0.502	-0.20	-0.47	0.09
		Control	57 vs 57	364	0.502	-0.16	-0.43	0.14
POMS2(TMD)	Baseline vs Load	Stress	57 vs 57	64	0.002	-0.92	-0.96	-0.86
		Control	57 vs 57	732	0.406	0.06	-0.23	0.35
	Baseline vs Recovery	Stress	57 vs 57	772	0.210	0.37	0.09	0.60
		Control	57 vs 57	710	0.525	0.03	-0.26	0.32
	Load vs Recovery	Stress	57 vs 57	1538	0.002	0.93	0.87	0.96
		Control	57 vs 57	606	0.845	-0.01	-0.30	0.28

STAI (State Anxiety)	Baseline vs Load	Stress	57 vs 57	68	0.002	-0.91	-0.95	-0.85
		Control	57 vs 57	556	0.181	-0.19	-0.46	0.10
	Baseline vs Recovery	Stress	57 vs 57	947	0.503	0.37	0.09	0.60
		Control	57 vs 57	767	0.503	-0.04	-0.33	0.25
	Load vs Recovery	Stress	57 vs 57	1437	0.002	0.93	0.88	0.96
		Control	57 vs 57	835	0.503	0.21	-0.08	0.47

Note: r_{rb}: rank-biserial correlation coefficient. 95%CI: 95% confidence interval (CI) of r_{rb}. For each index, the Conover test was used to compare the Situation(Baseline), Situation(Load), and Situation(Recovery) in each group, and multiple comparison correction was performed using Holm method for comparison between these three conditions.

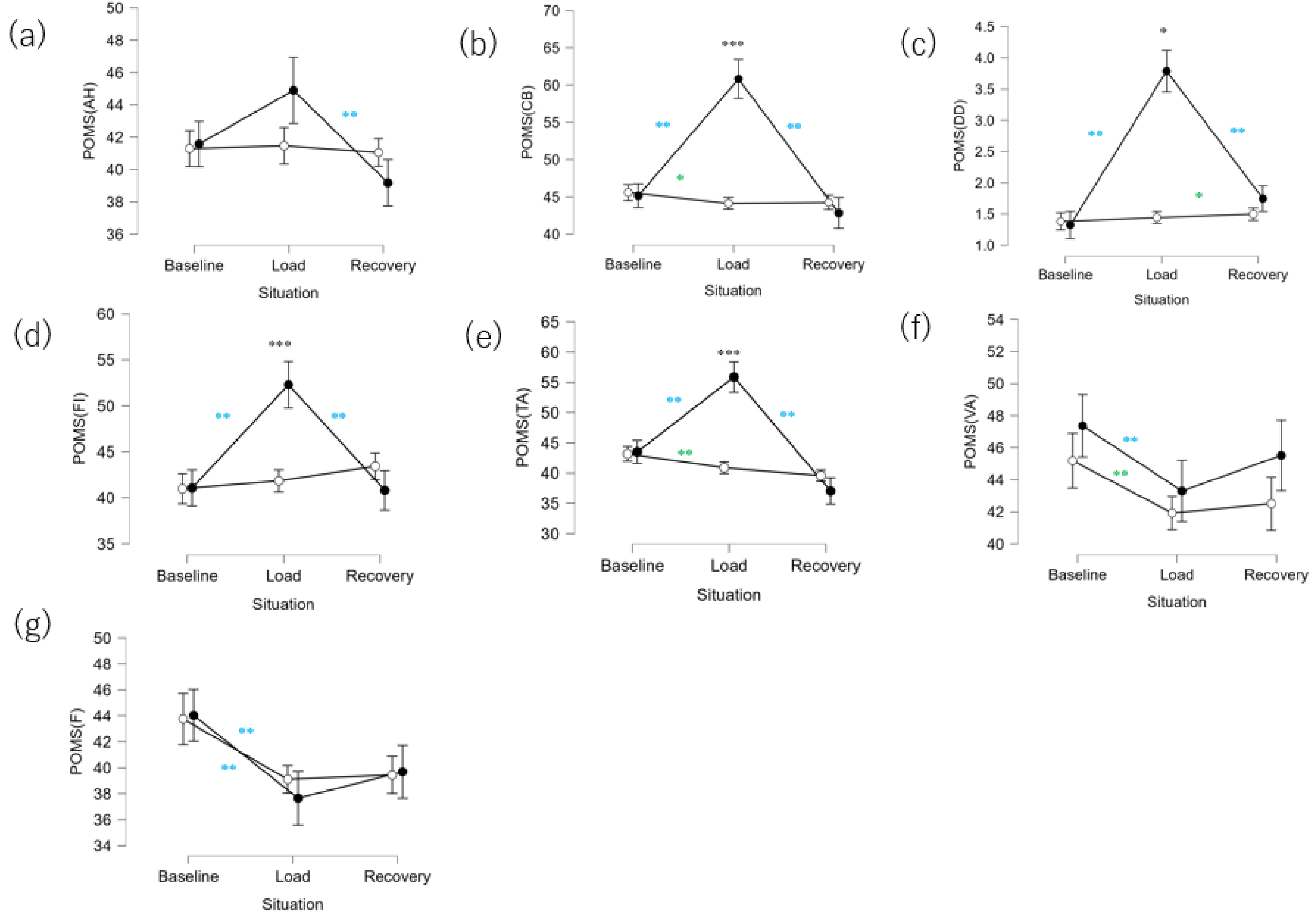
(a)
POMS(AH)
50
48
46
44
42
40
38
36
Baseline
Load
Recovery
Situation
(b)
POMS(CB)
70
65
60
55
50
45
40
Baseline
Load
Recovery
Situation
(c)
POMS(DD)
4.5
4.0
3.5
3.0
2.5
2.0
1.5
1.0
Baseline
Load
Recovery
Situation
(d)
POMS(FI)
60
55
50
45
40
35
Baseline
Load
Recovery
Situation
(e)
POMS(TA)
65
60
55
50
45
40
35
30
Baseline
Load
Recovery
Situation
(f)
POMS(VA)
54
52
50
48
46
44
42
40
Baseline
Load
Recovery
Situation
(g)
POMS(F)
50
48
46
44
42
40
38
36
34
Baseline
Load
Recovery
Situation

Figure S1. Inter-group comparisons for each of the items (POMS2: Anger- Hostility [AH], Confusion-Bewilderment [CB], Depression-Dejection [DD], Fatigue-Inertia [FI], Tension-Anxiety [TA], Vigour- Activity [VA], and Friendliness [F]) in each Situation. ● indicates the Stress group and ○ indicates the Control group. (a) POMS2 (AH); (b) POMS2 (CB); (c) POMS2 (DD); (d) POMS2 (FI); (e) POMS 2(TA); (f) POMS2 (VA); (g) POMS2 (F). $***p < 0.001$, $**p < 0.01$, $*p < 0.05$; Comparisons between Situations in the Stress and Control groups are shown in black; the comparison between Baseline and Load and between Load and Recovery in the Stress group are shown in blue; the comparison between Baseline and Load, and between Load and Recovery in the Control group is shown in green.

Table S4. Odds ratio of logistic regression model (H_0, H_1) with difference between groups as the response variable (Load)

	**H_0 model**			**H_1 model**		
	**Odds Ratio**	**95% Confidence interval (odds ratio scale)**		**Odds Ratio**	**95% Confidence interval (odds ratio scale)**	
		**Lower bound**	**Upper bound**		**Lower bound**	**Upper bound**
**Coefficients**						
**Intercept**	8.01×10^{-6}	0.00	0.01	8.66×10^{-5}	0.00	0.22
**SDPP Load**	-	-	-	4.83	2.27	10.26
**HR Load**	1.08	1.03	1.14	1.03	0.97	1.09
**LF/HF Load**	1.13	0.90	1.42	1.08	0.82	1.42
**HF Load**	1.00	1.00	1.01	1.00	1.00	1.01
**Cortisol Load**	1.37	1.12	1.67	1.43	1.10	1.85
**Age**	1.07	1.00	1.13	1.05	0.98	1.13
**Gender**	0.56	0.20	1.59	0.49	0.12	2.01
**BMI**	1.02	0.90	1.16	0.96	0.80	1.15

Note: The null model (H_0) included HR, LF/HF, HF, cortisol, age, gender, and BMI as explanatory variables, and the alternative model (H_1) included SDPP in addition to the explanatory variables in the null model. The sample sizes for each are: H_0 model = 102, H_1 model = 102.

Table S5. Comparison of logistic regression models (H_0, H_1) with difference between groups as the response variable and the results of estimated coefficients (Baseline)

	H_0 model			H_1 model		
	**Estimate**		***SE***	**Estimate**		***SE***
**Coefficients**						
**Intercept**	0.28	**	2.63	0.27	**	2.68
**SDPP Baseline**	-		-	-0.01		0.31
**HR Baseline**	-0.03		0.02	-0.03		0.02
**LF/HF Baseline**	-0.02		0.13	-0.02		0.13
**HF Baseline**	0.00		0.00	0.00		0.00
**Cortisol Baseline**	0.02		0.07	0.02		0.07
**Age**	0.02		0.03	0.02		0.03
**Gender**	-0.14		0.44	-0.15		0.45
**BMI**	0.04		0.06	0.04		0.06
**Model Summary**						
**Deviance**	134.48			134.48		
**AIC**	150.48			152.48		
**BIC**	171.48			176.11		
df	94			93		
X^2	-			0.00		
p	-			0.986		

		2.41×10^{-6}
**McFadden *R²***	-	
**Performance**		
**Accuracy**	0.55	0.55
**AUC**	0.61	0.61
**Sensitivity**	0.56	0.56
**Specificity**	0.54	0.54

Note: The null model (H_0) included HR, LF/HF, HF, cortisol, age, gender, and BMI as explanatory variables, and the alternative model (H_1) included SDPP in addition to the explanatory variables in the null model. *SE* = standard error; AIC = Akaike information criterion; BIC = Bayesian information criterion. ****p* < 0.001, ***p* < 0.01, **p* < 0.05. The sample sizes for each are as follows: H_0 model = 102, H_1 model = 102.

Table S6. Odds ratio of logistic regression models (H_0, H_1) with difference between groups as the response variable (Baseline)

	H_0 model			H_1 model		
	Odds Ratio	95% Confidence interval (odds ratio scale)		Odds Ratio	95% Confidence interval (odds ratio scale)	
		Lower bound	Upper bound		Lower bound	Upper bound
**Coefficients**						
**Intercept**	1.32	0.01	228.00	1.31	0.01	250.70
**SDPP Baseline**	-	-	-	0.99	0.54	1.84
**HR Baseline**	0.97	0.93	1.02	0.97	0.93	1.02
**LF/HF Baseline**	0.98	0.76	1.26	0.98	0.76	1.27
**HF Baseline**	1.00	1.00	1.00	1.00	1.00	1.00
**Cortisol Baseline**	1.02	0.89	1.16	1.02	0.89	1.16
**Age**	1.02	0.97	1.07	1.02	0.96	1.07
**Gender**	0.87	0.37	2.04	0.87	0.36	2.07
**BMI**	1.04	0.92	1.17	1.04	0.92	1.17

Note: The null model (H_0) included HR, LF/HF, HF, cortisol, age, gender, and BMI as explanatory variables, and the alternative model (H_1) included SDPP in addition to the explanatory variables in the null model. The sample sizes for each are as follows: H_0 model = 102, H_1 model = 102.

Table S7. Comparisons of logistic regression models (H_0, H_1) with difference between groups as the response variable and the results of estimated coefficients (Recovery)

	**Ho model**			**H1 model**		
	**Estimate**		***SE***	**Estimate**		***SE***
**Coefficients**						
**Intercept**	-2.83	**	2.96	-2.20		3.01
**SDPP Recovery**	-		-	0.53		0.29
**HR Recovery**	0.00		0.03	-0.02		0.03
**LF/HF Recovery**	0.14		0.11	0.14		0.12
**HF Recovery**	0.00		0.00	0.00		0.00
**Cortisol Recovery**	0.33	**	0.11	0.34	**	0.11
**Age**	0.03		0.03	0.02		0.03
**Gender**	-0.22		0.47	-0.25		0.48
**BMI**	0.00		0.06	-0.01		0.07
**Model Summary**						
**Deviance**	120.89			117.25		
**AIC**	136.89			135.25		
**BIC**	157.81			158.79		
df	93			92		
X^2	-			3.64		
p	-			0.057		
**McFadden** R^2	-			0.03		

**Performance**		
**Accuracy**	0.69	0.68
**AUC**	0.74	0.77
**Sensitivity**	0.72	0.74
**Specificity**	0.66	0.62

Note: The null model (H_0) included HR, LF/HF, HF, cortisol, age, gender, and BMI as explanatory variables, and the alternative model (H_1) included SDPP in addition to the explanatory variables in the null model. *SE* = standard error; AIC = Akaike information criterion; BIC = Bayesian information criterion. ***$p < 0.001$, **$p < 0.01$, *$p < 0.05$. Sample sizes for each are as follows: H_0 model = 101, H_1 model = 101.

Table S8. Odds ratio of logistic regression models (H_0, H_1) with difference between groups as the response variable (Recovery)

	**H_0 model**			**H_1 model**		
		**95% Confidence interval (odds ratio scale)**			**95% Confidence interval (odds ratio scale)**	
	**Odds Ratio**	**Lower bound**	**Upper bound**	**Odds Ratio**	**Lower bound**	**Upper bound**
**Coefficients**						
**Intercept**	0.06	0.00	19.33	0.11	0	40.13
**SDPP Recovery**	-		-	1.70	0.97	2.98
**HR Recovery**	1.00	0.95	1.05	0.98	0.93	1.03
**LF/HF Recovery**	1.15	0.92	1.44	1.15	0.92	1.45
**HF Recovery**	1.00	1.00	1.00	1.00	1.00	1.00
**Cortisol Load**	1.39	1.13	1.71	1.41	1.15	1.73
**Age**	1.03	0.98	1.08	1.02	0.97	1.08
**Gender**	0.80	0.32	2.01	0.78	0.30	2.00
**BMI**	1.00	0.88	1.13	0.99	0.87	1.13

Note: The null model (H_0) included HR, LF/HF, HF, cortisol, age, gender, and BMI as explanatory variables, and the alternative model (H_1) included SDPP in addition to the explanatory variables in the null model. Sample sizes for each are as follows: H_0 model = 101, H_1 model = 101.

Table S9. Comparison of logistic regression model with difference between groups as the response variable excluding participants (16

participants) with any of the following: smoking, medication, poor health status, or chronic illness and the results of the estimated coefficients (Load)

	H_0 model			H_1 model		
	**Estimate**		***SE***	**Estimate**		***SE***
**Coefficients**						
**Intercept**	-11.84	**	3.76	-10.19	*	4.41
**SDPP Load**	-		-	1.56	***	0.40
**HR Load**	0.08	**	0.03	0.04		0.03
**LF/HF Load**	0.08		0.13	0.01		0.15
**HF Load**	0.00		0.00	0.00		0.00
**Cortisol Load**	0.27	**	0.10	0.33	*	0.13
**Age**	0.06		0.03	0.05		0.04
**Gender**	-0.54		0.56	-0.72		0.77
**BMI**	0.04		0.07	-0.03		0.09
**Model Summary**						
**Deviance**	93.54			62.05		
**AIC**	109.54			80.05		
**BIC**	129.36			102.34		
df	80			79		
X^2	-			31.49		
p	-			< .001		
**McFadden** R^2	-			0.337		
**Performance**						

**Accuracy**	0.77	0.85
**AUC**	0.80	0.92
**Sensitivity**	0.76	0.89
**Specificity**	0.79	0.81

Note: The null model (H_0) included HR, LF/HF, HF, cortisol, age, gender, and BMI as explanatory variables, and the alternative model (H_1) included SDPP in addition to the explanatory variables in the null model. *SE* = standard error; AIC = Akaike information criterion; BIC = Bayesian information criterion. ***$p < 0.001$, **$p < 0.01$, *$p < 0.05$. Sample sizes for each are as follows: H_0 model = 88, H_1 model = 88.

Table S10. Odds ratio of logistic regression models (H_0, H_1) excluding 16 participants with any of the following: smoking, medication,

poor health, or chronic illness with difference between groups as the response variable (Load)

	H_0 model			H_1 model		
	Odds Ratio	95% Confidence interval (odds ratio scale)		Odds Ratio	95% Confidence interval (odds ratio scale)	
		Lower bound	Upper bound		Lower bound	Upper bound
**Coefficients**						
**Intercept**	7.20×10^{-6}	0.00	0.01	3.74×10^{-5}	0	0.21
**SDPP Load**	-		-	4.75	2.16	10.42
**HR Load**	1.08	1.03	1.14	1.04	0.97	1.11
**LF/HF Load**	1.08	0.84	1.40	1.01	0.75	1.34
**HF Load**	1.00	1.00	1.01	1.00	1.00	1.01
**Cortisol Load**	1.31	1.08	1.58	1.39	1.07	1.81
**Age**	1.06	1.00	1.13	1.05	0.98	1.14
**Gender**	0.58	0.20	1.73	0.49	0.11	2.21
**BMI**	1.05	0.92	1.19	0.97	0.81	1.17

Note: The null model (H_0) included HR, LF/HF, HF, cortisol, age, gender, and BMI as explanatory variables, and the alternative model (H_1) included SDPP in addition to the explanatory variables in the null model. Sample size for each is as follows: H_0 model = 88, H_1 model = 88.

Table S11. Comparison of the multilevel analysis model with POMS2 (AH: anger-hostility) as the response variable and the results of the estimated coefficients

	H_0 model			H_1 model		
	**Estimate**		***SE***	**Estimate**		***SE***
**Coefficients**						
**Intercept**	41.28	***	0.75	41.28	***	0.75
**SDPP**	-		-	0.04		0.84
**HR**	2.12	***	0.61	2.09	*	0.86
**LF/HF**	0.00	***	0.58	-0.01		0.60
**HF**	0.91		0.57	0.90		0.61
**Cortisol**	0.55		0.57	0.54		0.57
**Model Summary**						
**Deviance**	1065.27			1065.26		
**AIC**	1079.27			1081.26		
**BIC**	1100.61			1105.66		
df	7			8		
**log Lik.**	-532.63			-532.63		
X^2	-			0.00		
p	-			0.964		

Note: The null model (H_0) included HR, LF/HF, HF, and cortisol as explanatory variables, and the alternative model (H_1) included SDPP in addition to the explanatory variables in the null model. Standardizing scores (subtracting the mean values of Baseline, Load, and Recovery for each participant from each value, which was then divided by the standard deviation) were performed for explanatory variables. *SE* = standard error; AIC = Akaike information criterion; BIC = Bayesian information criterion; log Lik. = log-likelihood ratio. ***$p < 0.001$, **$p < 0.01$, *$p < 0.05$. The sample sizes for each are as follows: H_0 model = 52, H_1 model = 52.

Table S12. Comparison of the multilevel analysis model with POMS 2(CB: Confusion-Bewilderment) as the response variable and the results of the estimated coefficients

	H_0 model			H_1 model		
	**Estimate**		***SE***	**Estimate**		***SE***
**Coefficients**						
**Intercept**	49.51	***	1.15	49.51	***	1.15
**SDPP**	-		-	4.44	***	1.15
**HR**	6.30	***	0.88	3.13	**	1.16
**LF/HF**	1.14		0.85	0.42		0.81
**HF**	1.48		0.82	0.38		0.82
**Cortisol**	1.76		0.82	1.33		0.78
**Model Summary**						
**Deviance**	1186.57			1172.53		
**AIC**	1200.57			1188.53		
**BIC**	1221.92			1212.93		
df	7			8		
**log Lik.**	-593.29			-586.27		
X^2	-			14.04		
p	-			< .001		

Note: The null model (H_0) included HR, LF/HF, HF, and cortisol as explanatory variables, and the alternative model (H_1) included SDPP in addition to the explanatory variables in the null model. Standardizing scores (subtracting the mean values of Baseline, Load, and Recovery for each participant from each value, which was then divided by the standard deviation) were performed for explanatory variables. *SE* = standard error; AIC = Akaike information criterion; BIC = Bayesian information criterion; log Lik. = log-likelihood ratio. ***$p < 0.001$, **$p < 0.01$, *$p < 0.05$. The sample sizes for each are as follows: H_0 model = 52, H_1 model = 52.

Table S13. Comparison of multilevel analysis models with POMS2 (DD: Depression-Dejection) as the response variable and results of estimated coefficients.

	H_0 model			H_1 model		
	Estimate		*SE*	Estimate		*SE*
**Coefficients**						
**Intercept**	47.51	**	1.16	47.51	***	1.16
**SDPP**	-		-	2.13	*	0.93
**HR**	1.93	**	0.68	0.41		0.94
**LF/HF**	0.30		0.66	-0.04		0.66
**HF**	0.82		0.64	0.29		0.66
**Cortisol**	1.07		0.64	0.86		0.63
**Model Summary**						
**Deviance**	1134.31			1129.14		
**AIC**	1148.31			1145.14		
**BIC**	1169.65			1169.54		
df	7			8		
**log Lik.**	-567.15			-564.57		
X^2	-			5.16		
p	-			0.023		

Note: The null model (H_0) included HR, LF/HF, HF, and cortisol as explanatory variables, and the alternative model (H_1) included SDPP in addition to the explanatory variables in the null model. Standardizing scores (subtracting the mean values of Baseline, Load, and Recovery for each participant from each value, which was then divided by the standard deviation) were performed for explanatory variables. *SE* = standard error; AIC = Akaike information criterion; BIC = Bayesian information criterion; log Lik. = log-likelihood ratio. ***$p < 0.001$, **$p < 0.01$, *$p < 0.05$. The sample sizes for each are as follows: H_0 model = 52, H_1 model = 52.

Table S14. Comparison of the multilevel analysis model with POMS2 (FI: Fatigue-Inertia) as the response variable and the results of the estimated coefficients

	H_0 model			H_1 model		
	**Estimate**		***SE***	**Estimate**		***SE***
**Coefficients**						
**Intercept**	44.53	***	1.27	44.53	***	1.27
**SDPP**	-		-	2.77	*	1.12
**HR**	4.60	***	0.83	2.62	*	1.13
**LF/HF**	1.17		0.79	0.72		0.79
**HF**	1.15		0.77	0.46		0.80
**Cortisol**	0.88		0.77	0.61		0.76
**Model Summary**						
**Deviance**	1184.40			1178.43		
**AIC**	1198.40			1194.43		
**BIC**	1219.75			1218.83		
df	7			8		
**log Lik.**	-592.20			-589.22		
X^2	-			5.97		
p	-			0.015		

Note: The null model (H_0) included HR, LF/HF, HF, and cortisol as explanatory variables, and the alternative model (H_1) included SDPP in addition to the explanatory variables in the null model. Standardizing scores (subtracting the mean values of Baseline, Load, and Recovery for each participant from each value, which was then divided by the standard deviation) were performed for explanatory variables. *SE* = standard error; AIC = Akaike information criterion; BIC = Bayesian information criterion; log Lik. = log-likelihood ratio. ***$p < 0.001$, **$p < 0.01$, *$p < 0.05$. The sample sizes for each are as follows: H_0 model = 52, H_1 model = 52.

Table S15. Comparison of the multilevel analysis model with POMS2 (TA: Tension-Anxiety) as the response variable and the results of the estimated coefficients

	H_0 model			H_1 model		
	Estimate		*SE*	Estimate		*SE*
**Coefficients**						
**Intercept**	45.25	***	1.18	45.25	***	1.18
**SDPP**	-		-	2.74	*	1.24
**HR**	6.51	***	0.91	4.56	***	1.25
**LF/HF**	-0.06		0.87	-0.50		0.88
**HF**	0.94		0.85	0.26		0.89
**Cortisol**	1.90		0.85	1.63		0.84
**Model Summary**						
**Deviance**	1195.54			1190.74		
**AIC**	1209.54			1206.74		
**BIC**	1230.89			1231.14		
df	7			8		
**log Lik.**	-597.77			-595.37		
X^2	-			4.80		
p	-			0.028		

Note: The null model (H_0) included HR, LF/HF, HF, and cortisol as explanatory variables, and the alternative model (H_1) included SDPP in addition to the explanatory variables in the null model. Standardizing scores (subtracting the mean values of Baseline, Load, and Recovery for each participant from each value, which was then divided by the standard deviation) were performed for explanatory variables. *SE* = standard error; AIC = Akaike information criterion; BIC = Bayesian information criterion; log Lik. = log-likelihood ratio. ***$p < 0.001$, **$p < 0.01$, *$p < 0.05$. The sample sizes for each are as follows: H_0 model = 52, H_1 model = 52.

Table S16. Comparison of the multilevel analysis model with POMS2 (VA: Vigour-Activity) as the response variable and the results of the estimated coefficients

	H_0 model			H_1 model		
	**Estimate**		***SE***	**Estimate**		***SE***
**Coefficients**						
**Intercept**	44.46	***	1.27	44.46	***	1.27
**SDPP**	-		-	-1.49		0.96
**HR**	-1.74	*	0.70	-0.67		0.98
**LF/HF**	-0.84		0.67	-0.59		0.68
**HF**	-0.36		0.66	0.01		0.69
**Cortisol**	0.24		0.65	0.39		0.65
**Model Summary**						
**Deviance**	1149.55			1147.18		
**AIC**	1163.55			1163.18		
**BIC**	1184.90			1187.58		
df	7			8		
**log Lik.**	-574.78			-573.59		
X^2	-			2.38		
p	-			0.123		

Note: The null model (H_0) included HR, LF/HF, HF, and cortisol as explanatory variables, and the alternative model (H_1) included SDPP in addition to the explanatory variables in the null model. Standardizing scores (subtracting the mean values of Baseline, Load, and Recovery for each participant from each value, which was then divided by the standard deviation) were performed for explanatory variables. *SE* = standard error; AIC = Akaike information criterion; BIC = Bayesian information criterion; log Lik. = log-likelihood ratio. ***$p < 0.001$, **$p < 0.01$, *$p < 0.05$. The sample sizes for each are as follows: H_0 model = 52, H_1 model = 52.

Table S17. Comparison of the multilevel analysis model with POMS2 (F: Friendliness) as the response variable and the results of the estimated coefficients

	H$_0$ model			H$_1$ model		
	**Estimate**		***SE***	**Estimate**		***SE***
**Coefficients**						
**Intercept**	39.31	***	1.41	39.31	***	1.41
**SDPP**	-		-	-1.59		1.03
**HR**	-1.31		0.75	-0.18		1.04
**LF/HF**	-1.35		0.72	-1.10		0.73
**HF**	-0.84		0.70	-0.45		0.74
**Cortisol**	-0.74		0.70	-0.58		0.70
**Model Summary**						
**Deviance**	1173.37			1171.01		
**AIC**	1187.37			1187.01		
**BIC**	1208.72			1211.41		
df	7			8		
**log Lik.**	-586.69			-585.50		
X^2	-			2.37		
p	-			0.124		

Note: The null model (H$_0$) included HR, LF/HF, HF, and cortisol as explanatory variables, and the alternative model (H$_1$) included SDPP in addition to the explanatory variables in the null model. Standardizing scores (subtracting the mean values of Baseline, Load, and Recovery for each participant from each value, which was then divided by the standard deviation) were performed for explanatory variables. *SE* = standard error; AIC = Akaike information criterion; BIC = Bayesian information criterion; log Lik. = log-likelihood ratio. ***$p < 0.001$, **$p < 0.01$, *$p < 0.05$. The sample sizes are for each as follows: H$_0$ model = 52, H$_1$ model = 52.

Table S18. Comparison of multilevel analysis models with STAI (State Anxiety) excepting HRV as explanatory variable and the results of estimated coefficients

	H0 model			H1 model		
	**Estimate**		***SE***	**Estimate**		***SE***
**Coefficients**						
**Intercept**	45.48	***	0.96	45.48	***	0.96
**SDPP**	-		-	2.51	*	0.97
**HR**	5.50	***	0.74	3.86	***	0.96
**Cortisol**	0.99		0.74	0.63		0.73
**Model Summary**						
**Deviance**	1252.84			1246.36		
**AIC**	1262.84			1258.36		
**BIC**	1278.46			1277.10		
df	5			6		
**log Lik.**	-626.42			-623.18		
X^2	-			6.48		
p	-			0.01		

Note: The null model (H_0) included HR and cortisol as explanatory variable, and the alternative model (H_1) included SDPP in addition to the explanatory variables in the null model. Standardizing scores (subtracting the mean values of Baseline, Load, and Recovery for each participant from each value, which was then divided by the standard deviation) were performed for explanatory variables. *SE* = standard error; AIC = Akaike information criterion; BIC = Bayesian information criterion; log Lik. = log-likelihood ratio. ***$p < 0.001$, **$p < 0.01$, *$p < 0.05$. The sample sizes for each are as follows: H_0 model = 52, H_1 model = 52.

Table S19. Comparison of the multilevel analysis model with the STAI (state anxiety) including SDPP, HR and cortisol as the response variables, with adjustment of the number of cases (n=53) excluding missing data of participants for whom LF/HF and HF were not

available, and the results of estimated coefficients

	H_0 model			H_1 model		
	**Estimate**		***SE***	**Estimate**		***SE***
**Coefficients**						
**Intercept**	45.58	***	1.03	45.58	***	1.03
**SDPP**	-			1.92		1.06
**HR**	5.78	***	0.77	4.46	***	1.05
**Cortisol**	1.05		0.77	0.78		0.77
**Model Summary**						
**Deviance**	1163.89			1160.67		
**AIC**	1173.89			1172.67		
**BIC**	1189.14			1190.97		
df	5			6		
**log Lik.**	-581.95			-580.34		
X^2	-			3.220		
p	-			0.07274		

Note: The null model (H_0) included HR and cortisol as explanatory variable, and the alternative model (H_1) included SDPP in addition to the explanatory variables in the null model. Missing data of participants for whom LF/HF and HF were not available were excluded from both H_0 and H_1, and the analysis was performed adjusting the number of cases. Standardizing scores (subtracting the mean values of Baseline, Load, and Recovery for each participant from each value, which was then divided by the standard deviation) were performed for explanatory variables. *SE* = standard error; AIC = Akaike information criterion; BIC = Bayesian information criterion; log Lik. = log-likelihood ratio. ***$p < 0.001$, **$p < 0.01$, *$p < 0.05$. The sample sizes for each are: H_0 model = 52, H_1 model = 52.
